# StackER: a novel SMILES-based stacked approach for the accelerated and efficient discovery of ERα and ERβ antagonists

**DOI:** 10.1038/s41598-023-50393-w

**Published:** 2023-12-27

**Authors:** Nalini Schaduangrat, Nutta Homdee, Watshara Shoombuatong

**Affiliations:** Center for Research Innovation and Biomedical Informatics, Faculty of Medical Technology, Mahidol University, Bangkok, 10700 Thailand

**Keywords:** Cheminformatics, Data mining, Machine learning

## Abstract

The role of estrogen receptors (ERs) in breast cancer is of great importance in both clinical practice and scientific exploration. However, around 15–30% of those affected do not see benefits from the usual treatments owing to the innate resistance mechanisms, while 30–40% will gain resistance through treatments. In order to address this problem and facilitate community-wide efforts, machine learning (ML)-based approaches are considered one of the most cost-effective and large-scale identification methods. Herein, we propose a new SMILES-based stacked approach, termed StackER, for the accelerated and efficient identification of ERα and ERβ inhibitors. In StackER, we first established an up-to-date dataset consisting of 1,996 and 1,207 compounds for ERα and ERβ, respectively. Using the up-to-date dataset, StackER explored a wide range of different SMILES-based feature descriptors and ML algorithms in order to generate probabilistic features (PFs). Finally, the selected PFs derived from the two-step feature selection strategy were used for the development of an efficient stacked model. Both cross-validation and independent tests showed that StackER surpassed several conventional ML classifiers and the existing method in precisely predicting ERα and ERβ inhibitors. Remarkably, StackER achieved MCC values of 0.829–0.847 and 0.712–0.786 in terms of the cross-validation and independent tests, respectively, which were 5.92–8.29 and 1.59–3.45% higher than the existing method. In addition, StackER was applied to determine useful features for being ERα and ERβ inhibitors and identify FDA-approved drugs as potential ERα inhibitors in efforts to facilitate drug repurposing. This innovative stacked method is anticipated to facilitate community-wide efforts in efficiently narrowing down ER inhibitor screening.

## Introduction

Estrogen receptors (ERs) play a crucial role in the initiation and advancement of breast cancer, a prevalent malignancy that affects millions worldwide^1^. Breast cancer is a diverse ailment, and its different subcategories are frequently identified by whether ERs are present or absent^2^ ERs are proteins located in breast cells that engage with the hormone estrogen, which is a vital regulator of numerous physiological processes, including the development and upkeep of breast tissue^3^. In this context, ERs serve as molecular switches that can either promote or hinder the growth and proliferation of breast cancer cells, depending on the presence or absence of estrogen.

There are two primary estrogen receptors: ERα and ERβ. ERα is predominantly situated in breast tissue and can also be found in the uterus, ovaries, and other reproductive organs. When estrogen activates ERα, it is associated with the stimulation of cell growth and replication, which is essential for the development and maintenance of breast tissue. In contrast, ERβ is found in breast tissue, although in smaller quantities compared to ERα, and it is also commonly distributed in various other tissues throughout the body, including the prostate, colon, and bone^4^. The function of ERβ is more complex and not as well-understood as that of ERα. However, recent research has emerged emphasizing ERβ's anti-cancer properties and its potential as a predictor of treatment effectiveness, irrespective of the presence of ERα^5,6^. Grasping the role of ERs in breast cancer is of great importance in both clinical practice and scientific exploration. This comprehension has paved the way for the development of tailored treatments specifically designed to address ER-positive breast cancers, resulting in improved treatment outcomes and overall patient survival rates. These therapeutic strategies involve substances that mitigate the effects of estradiol by competitively binding to ER, such as selective estrogen receptor modulators (SERMs, like tamoxifen), which decrease the levels of natural estrogens, such as aromatase inhibitors (e.g., letrozole, anastrozole, and exemestane), or a selective estrogen receptor degrader (SERD, like fulvestrant), which completely counter and degrade ER^7^. However, around 15–30% of those affected do not see benefits from the usual treatments owing to the innate resistance mechanisms, and during treatment, around 30–40% will acquire resistance^8,9^. Therefore, the development of treatment resistance is a significant factor leading to unfavorable outcomes and remains a substantial challenge in the management of ER-positive breast cancer.

To address the problem of resistance, researchers are exploring various computer-assisted approaches for drug design. These methods include quantitative structure–activity relationship (QSAR)^10–12^, machine learning (ML)-based models^13–15^, deep learning (DL)-based models^16^, molecular docking^10,17,18^, molecular dynamic simulations^18,19^, and pharmacophore analysis^18^, among others. It's important to note that most of these research endeavors primarily focus on targeting ERα rather than ERβ^20^. To date, there is only one ML-based approach (named ERpred^21^) that is developed for predicting the effectiveness of inhibitors against ERα and ERβ. ERpred is a random forest-based model trained on 659 and 714 compounds for ERα and ERβ, respectively. Although ERpred provided reasonable prediction performance, there are three major issues that need to be addressed. Firstly, because the existing datasets used to develop ERpred contained a small number of compounds (Table 1), their predictive ability might be unsatisfactory for real-life applications. Secondly, ERpred did not conduct a comparative analysis among different ML classifiers and molecular descriptors in the identification of ERα and ERβ inhibitors. Lastly, ERpred was developed using only single ML algorithm and molecular descriptor. On the other hand, ensemble learning approach can automatically integrate several different ML classifiers to enhance the predictive performance.

**Table 1:** Comparison of training and independent test datasets used in ERpred and this study.

Subtype	Class	ERpred		This study	
		Training	Independent	Training	Independent
ERα	Active	283	70	916	229
	Inactive	245	61	680	171
	Total	528	131	1596	400
ERβ	Active	447	111	588	148
	Inactive	125	31	376	95
	Total	572	142	964	243

Keeping these issues in mind, we introduce StackER, a stacked ensemble learning approach for the accelerated and accurate identification of inhibitors against ERα and ERβ using SMILES information only. To obtain an accurate prediction model, first, we established an up-to-date dataset by collecting positive and negative samples from the ChEMBL database. Second, we investigated and evaluated variant ML models in predicting ERα and ERβ inhibitors by employing nine different types of SMILES-based feature descriptors (i.e., AP2D, AP2DC, FP4, FP4C, KR, KRC, MACCS, Pubchem, and RDK5) cooperating with eight popular ML algorithms (i.e., RF, generalized linear model (GLM), support vector machine (SVM), extreme gradient boosting (XGB), k-nearest neighbors (KNN), partial least squares regression (PLS), recursive partitioning and regression tree (rpart), and multi-layer perceptron (MLP)). Their predictive performances were obtained by performing the tenfold cross-validation and independent tests. In the meanwhile, all the ML classifiers were applied to generate probabilistic features (PFs). Finally, the optimal PFs were identified through a two-step feature selection method and used for the development of an efficient stacked model. Experimental results based on the cross-validation and independent tests showed that StackER can achieve a better overall performance as compared to several conventional ML classifiers and the existing method in precisely predicting inhibitors against ERα and ERβ. Furthermore, StackER was applied to identify important features for being ERα and ERβ inhibitors to be substructures with fluorine and nitrogen-containing and cyclohexanone derativatives, respectively, while our proposed model was used to identify FDA-approved drugs as potential ERα inhibitors in efforts to facilitate drug repurposing.

## Materials and methods

### Data collection and curation

The datasets for ERα and ERβ (CHEMBL206 and CHEMBL242, respectively) were obtained from the ChEMBL database^22^ (version 33, accessed on August 20, 2023). Initially, there were 15,446 compounds for ERα and 8979 compounds for ERβ in the dataset. In this study, we collected the IC50 bioactivity data for inhibitory activity against ERα and ERβ from the initial dataset, resulting in 5180 compounds for ERα and 3605 compounds for ERβ. These curated datasets underwent further pre-processing, which involved standardizing the chemical structure representations (SMILES), removing duplicates, and eliminating salt components. All of these pre-processing steps were carried out using the R programming language^23^. Then, we obtained the subsequent dataset consisting of 2532 and 1577 compounds for ERα and ERβ, respectively. To generate active and inactive compounds, we applied the same criteria as employed in previous studies^21,24–26^. As a result, we obtained actives and inactives (ERα and ERβ) of (1145 and 736) and (851 and 471), respectively. Finally, we randomly selected 80% of all compounds for each subtype to construct the training datasets, whereas the remaining compounds were used to construct the independent test datasets. The detail of the training and independent test datasets involved in this study is provided in Table 1.

### Descriptor extraction

For each compound, we generated multiple sets of fingerprint descriptors using the PaDEL-Descriptor software^27^ and RDKit (https://www.rdkit.org). Molecular fingerprints are widely employed in the field of cheminformatics because they effectively capture the structural characteristics of chemical compounds^28–30^. In this study, we considered nine different categories of molecular fingerprints, which include AP2D, AP2DC, KR, KRC, MACCS, Pubchem, FP4, FP4C, and RDK5^31–36^. A summary of these descriptor types is recorded in Table 2. In essence, we used the chemical structures represented in SMILES format as input to compute the fingerprint descriptors. Before the calculation of these descriptors, we standardized the tautomeric forms and removed any salt components. In total, we extracted eight molecular descriptors using the R programming environment (version 4.3.0^23^) and the RDK5 fingerprint descriptor was extracted using the Python programming environment^37^.

**Table 2:** Summary of nine molecular fingerprints used in this study.

Fingerprint	Abbreviation	#Feature	Description	Ref
2D atom pair	AP2D	780	Presence of atom pairs at various topological distances	^31^
2D atom pair count	AP2DC	780	Count of atom pairs at various topological distances	^31^
Klekota–Roth	KR	4,860	Presence of chemical substructures	^32^
Klekota–Roth count	KRC	4,860	Count of chemical substructures	^32^
MACCS	MACCS	166	Binary representation of chemical features defined by MACCS keys	^35^
Pubchem	Pubchem	881	Binary representation of substructures defined by PubChem	^34^
Substructure	FP4	307	Presence of SMARTS patterns for functional groups	^36^
Substructure count	FP4C	307	Count of SMARTS patterns for functional groups	^36^
RDK5	RDK5	2048	Binary representation of daylight-like substructures with path length 5	^33^

### Two-step feature selection strategy

From the viewpoint of classification, the feature selection procedure is an important step to exclude noisy features while improving performance. Herein, we applied a two-step feature selection method to determine *m* informative features to construct the final model. In the first step, RF method was used to assess the importance score of each feature. The RF method used herein was implemented in the R programming environment (version 4.3.1)^38^. Then, all the features were ranked according to their importance scores. The RF method is widely applied in various biological and chemical classification problems^21,24,39,40^. After obtaining the ranked features, we constructed *n* feature sets containing the *m* top-ranked important features ranging from top *m*_*start*_ to *m*_*end*_ with an interval of *s.* The values of *m*_*start*_, *m*_*end*_, *s*, and *n* depend on the feature dimension. In the second step, ML models were trained using all the *n* feature sets and their performance were assessed using the tenfold cross-validation test. The optimal feature set having the highest Matthews correlation coefficient (MCC) was utilized to construct the final model in this study.

### StackER framework

**Figure 1:**
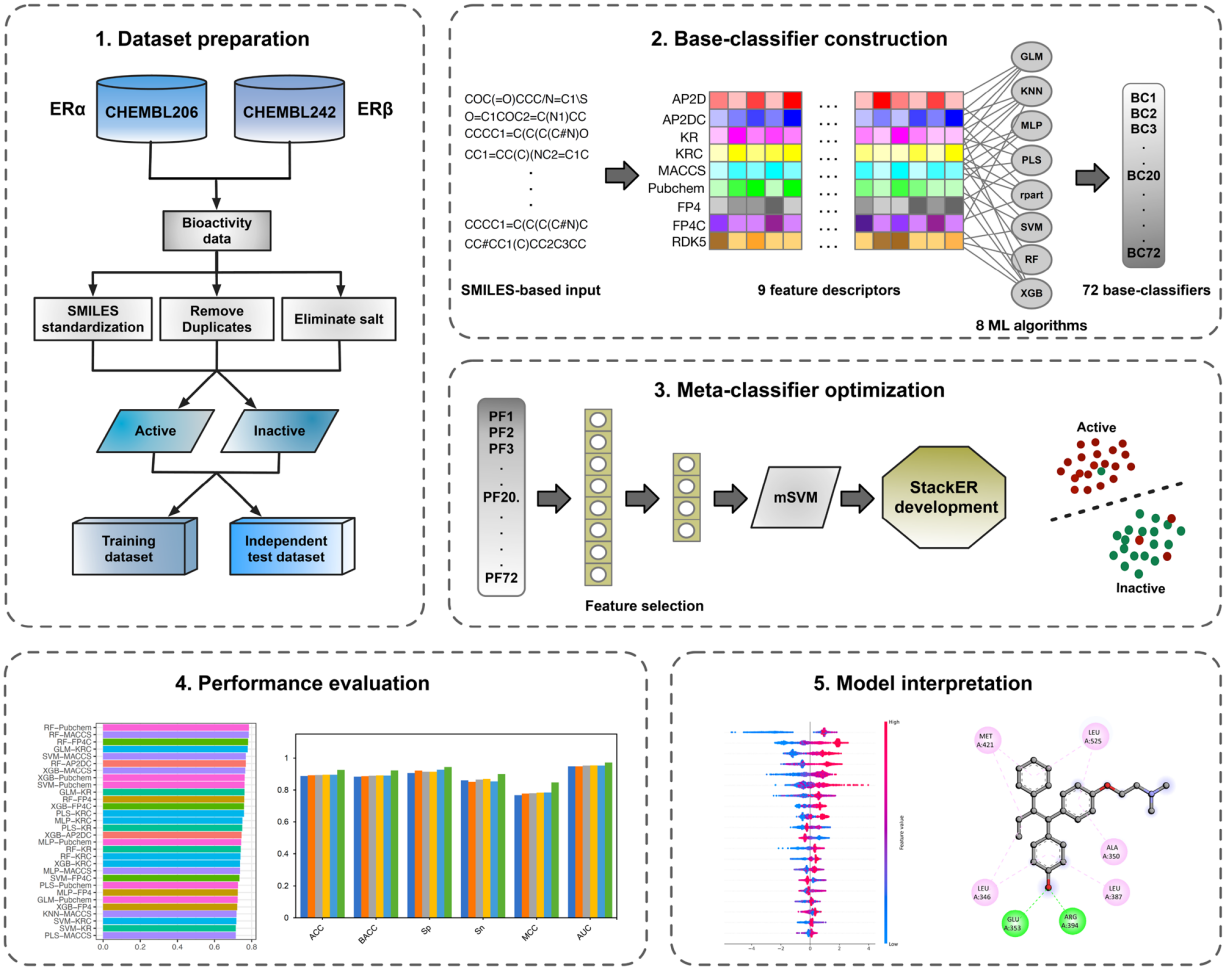
Workflow of the StackER development for identifying inhibitors against ERα and ERβ. This framework involves four primary steps, which include dataset preparation, base-classifier construction, meta-classifier optimization, and performance evaluation and model interpretation.

Stacking is a powerful ensemble learning strategy that allows the integration of the outputs of heterogynous prediction models as mean to construct a stacked model. Numerous studies have highlighted that the stacked models outperform single-based models in terms of high accuracy and low error. As shown in Fig. 1**,** the stacking strategy uses a two-layer learning framework, where the corresponding classifiers at each layer are referred as base-classifier and meta-classifier. In brief, the base-classifier is constructed using the original feature descriptors and used to generate PFs. Then, the PFs are considered as the input feature for the meta-classifier construction. A detailed description of the stacking strategy is provided in details as follows.

In the first-layer, we employed eight ML algorithms (i.e., GLM, MLP, KNN, RF, PLS, rpart, SVM and XGB) cooperating with nine molecular descriptors (i.e., AP2D, AP2DC, KR, KRC, FP4, FP4C, MACCS, Pubchem, and RDK5) to construct heterogeneous base-classifiers. As a result, we obtained a total of 72 base-classifiers, which were implemented based on the caret package for the R programming environment (version 4.3.1)^38^, their parameters were tuned using the grid optimization algorithm^24,26,41–43^ (**Supplementary Table S1**). After that, we employed these base-classifiers to generate PFs. The PF generation based on the stacking strategy is as following: (i) we used the tenfold cross-validation procedure to randomly divide the training dataset (D_TRN_) into 10 equal-sized subsets, where ${D}_{TRN}=\{{S}_{1}, {S}_{2},\dots ,{S}_{10}\}$; (ii) for the k^th^ iteration, we treated ${D}_{TRN}-{S}_{k}$ and ${S}_{k}$ as the current training and testing sets. The base-classifier trained with ${D}_{TRN}-{S}_{k}$ was used to generate the prediction output (${P}_{k}$); and we obtained 10 prediction outputs $\{{P}_{1}, {P}_{2},\dots ,{P}_{10}\}$ of D_TRN_. Then, the PF was obtained by averaging the 10 prediction outputs. Finally, in this layer, 72 PFs of all the 72 base-classifiers were obtained and represented with a 72-D probabilistic feature vector (APF). In the second layer, the meta-classifier was constructed using the SVM method (called mSVM) cooperated with APF. To optimize the performance of the meta-classifier, the two-step feature selection method was employed to determine a set of optimal PFs (called OPF). As a result, the values of *m*_*start*_, *m*_*end*_, *s*, and *n* are 5, 50, 5, and 14, respectively. The optimal feature set having the highest MCC was utilized to construct the final stacked models for the identification of inhibitors against ERα and ERβ. Moreover, we employed six well-known performance metrics, including MCC, area under the receiver operating characteristic (ROC) curve (AUC), accuracy (ACC), balanced accuracy (BACC), specificity (Sp), and sensitivity (Sn) to assess the performance of the proposed model and conventional ML models. The details of these six performance metrics are mentioned in the **Supplementary Information**.

### Case study and docking study of FDA-approved drugs

In this study, we obtained a library of FDA-approved small molecule drugs, consisting of 2,735 compounds, from the DrugBank database (version 5.1.10; released on January 4, 2023). After removing salt and inorganic compounds, as well as eliminating duplicate and disconnected SMILES representations and SMILES with explicit valence, the remaining number of compounds was reduced to 1,737. We then calculated molecular descriptors for all these compounds, which were used as input for prediction with our StackER model. The top fifteen compounds identified by our stack model were subsequently subjected to docking analysis, facilitating drug repurposing efforts. The target protein (PDB ID: 3ERT) was obtained from the Protein Data Bank (https://www.rcsb.org) and adjusted for docking. This optimization involved energy minimization in the SwissPDB Viewer^44^ and the addition of polar hydrogens and removal of water molecules in AutoDockTools version 1.5.7. Likewise, in order to ensure docking compatibility with AutoDock Vina^45^, ligands were prepared using AutoDockTools. Both the optimized protein and ligands were saved in pdbqt file formats. To enable accurate binding affinity calculations, we used the amino acid residues in the active site of the ERα protein to define a grid with dimensions of 50 × 40 × 48, with its center coordinates set at X = 29.621, Y = −0.545, Z = 26.455. The binding affinity of the ligands was determined by docking them inside the predetermined grid box of the target protein. The exhaustiveness was set to 32, and the energy range was set to 4, with the maximum energy difference between the best and worst binding mode not exceeding 3 kcal/mol. The binding potential of individual ligands can be represented by docking score or energy, where lower scores indicate higher binding affinity^46,47^. Finally, the analysis of the docked protein–ligand binding complexes was carried out using Discovery Studio software.

## Results and discussion

### Analysis of applicability domain

The applicability domain (AD) of a QSAR model delineates a region within the chemical space where the model is expected to provide accurate predictions^48^. To understand this, we employed t-distributed stochastic neighbor embedding (t-SNE) to visually represent the feature space associated with the nine molecular fingerprints. The visualizations in **Supplementary Figs. 1 and 2** depict the compounds from both the training and independent datasets, denoted in green and red, respectively, for ERα and ERβ. The AD boundary was established based on the characteristics of the training dataset, and compounds falling within this boundary are considered to be within the model's applicability domain. As seen in **Supplementary Figs. 1 and 2**, all nine molecular fingerprints for both protein subtypes exhibited overlapping chemical spaces between the training and independent datasets, indicating their suitability for the models developed in this study.

### Construction of StackER

In this section, we constructed different SVM-based meta-classifiers by taking advantages of our two new probabilistic feature vectors (i.e., APF and OPF) to provide more accurate ERα and ERβ inhibitors prediction. In addition, to improve the predictive performance, we used the two-step feature selection strategy to independently optimize the APF for each subtype. The two-step feature selection strategy determined 35 and 35 important PFs for developing SVM-based meta-classifiers for ERα and ERβ, respectively. Table 3 lists the performance evaluation results of four SVM-based meta-classifiers in terms of both the cross-validation and independent tests. In the case of ERα, OPF provided a better performance than APF in terms of BACC, Sn, and MCC on the training dataset, while the performance of OPF was comparable to APF in terms of BACC (0.894 versus 0.989) and MCC (0.786 versus 0.792). Impressively, OPF performed better than APF in terms of both the cross-validation and independent tests for ERβ subtype. To be specific, on the independent test dataset, the BACC, MCC, and AUC values of OPF were 0.849, 0.712, and 0.974, which were 4.02, 7.10, and 7.37%, respectively, higher than APF. Therefore, we applied the OPF to develop SVM-based meta-classifiers (called StackER) for ERα and ERβ in the following studies.

**Table 3:** Cross-validation and independent test results of different feature representations.

Subtype	Evaluation strategy	Feature	ACC	BACC	Sn	Sp	MCC	AUC
ERα	Cross-validation	APF	0.922	0.919	0.939	0.899	0.840	0.978
		OPF	0.925	0.922	0.945	0.899	0.847	0.973
	Independent test	APF	0.898	0.898	0.895	0.901	0.792	0.952
		OPF	0.895	0.894	0.900	0.889	0.786	0.973
ERβ	Cross-validation	APF	0.909	0.904	0.925	0.883	0.808	0.962
		OPF	0.918	0.916	0.925	0.907	0.829	0.974
	Independent test	APF	0.831	0.809	0.912	0.705	0.641	0.900
		OPF	0.864	0.849	0.919	0.779	0.712	0.974

### Stacking strategy contributes to performance improvement

**Figure 2:**
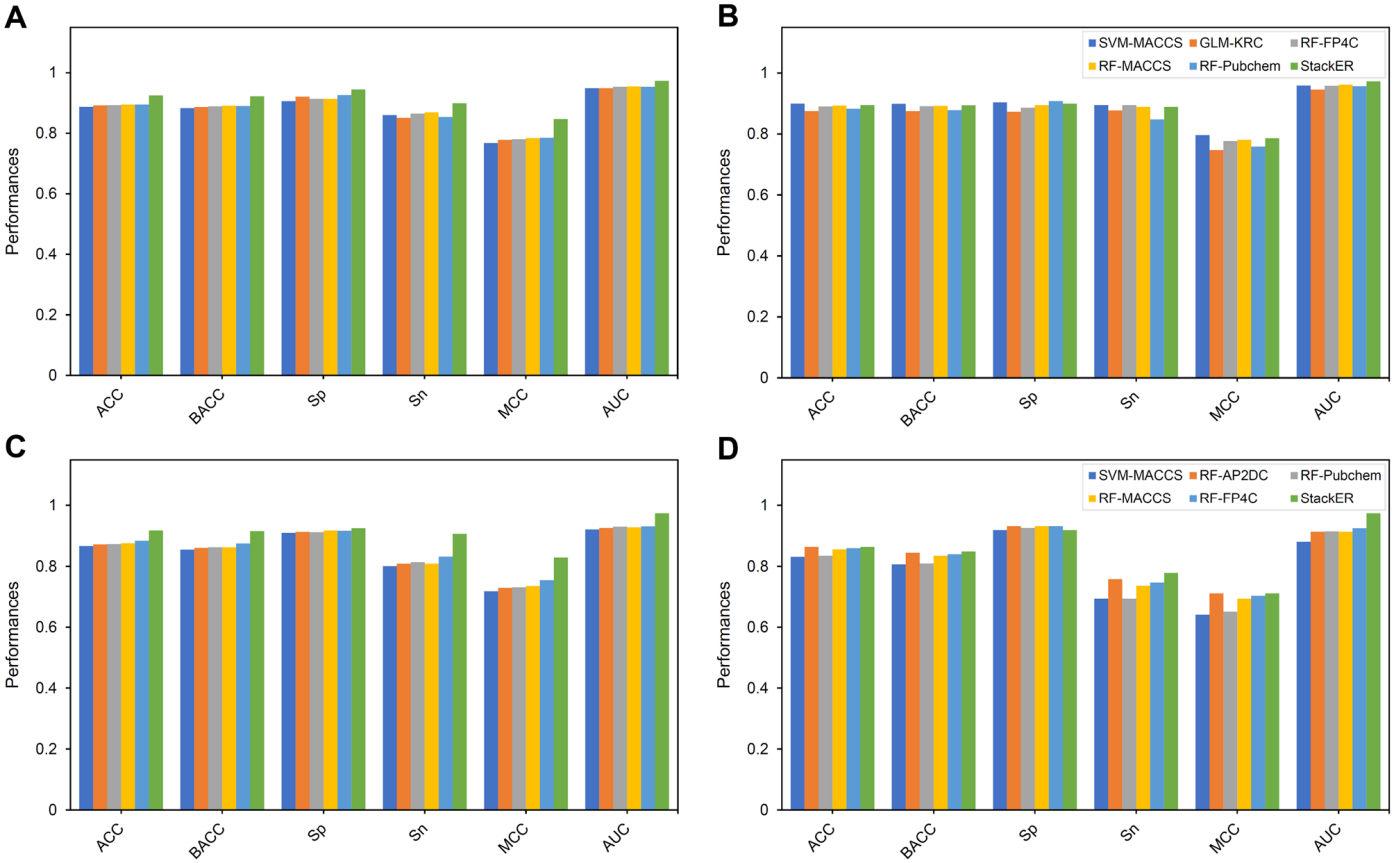
Performance comparison of StackER and top-five prediction models for ERα **(A,B)** and ERβ **(C,D)** subtypes assessed by the tenfold cross-validation **(A**,**C)** and independent tests **(B**,**D)**.

**Figure 3:**
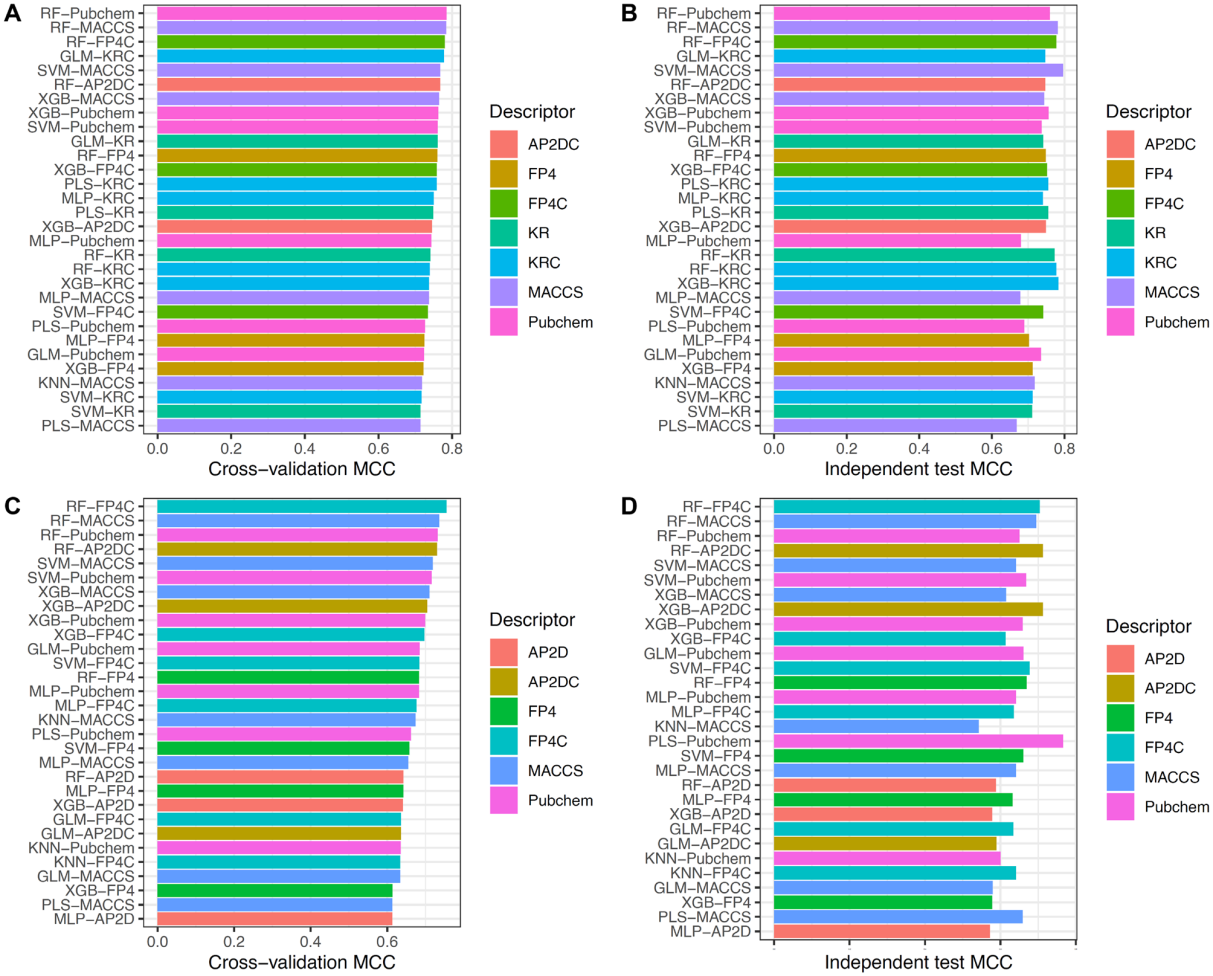
MCC values of top-30 ML classifiers for ERα **(A,B)** and ERβ **(C,D)** assessed by the tenfold cross-validation **(A**,**C)** and independent tests **(B**,**D)**.

To verify the necessity of the stacking strategy in this study, we compared the performance of StackER against conventional ML classifiers for predicting inhibitors against ERα and ERβ. As mentioned above, these ML classifiers were independently developed using 8 ML methods cooperating with 9 molecular descriptors for each subtype. The performance results of all the ML classifiers are summarized in **Supplementary Tables 2, 3, 4 and 5.** In addition, we selected 5 top-ranked ML classifiers having high cross-validation MCC for conducting a comparative analysis herein (Fig. 2). As shown in Fig. 3 and Tables 4 and 5**,** the 5 top-ranked ML classifiers for predicting inhibitors against ERα consist of RF-Pubchem, RF-MACCS, RF-FP4C, GLM-KRC, and SVM-MACCS with respective MCC values of 0.785, 0.784, 0.780, 0.778, and 0.768 (Table 4**)**, while the 5 top-ranked ML classifiers for predicting inhibitors against ERβ consist of RF-FP4C, RF-MACCS, RF-Pubchem, RF-AP2DC, SVM-MACCS with respective MCC values of 0.755, 0.736, 0.732, 0.730, and 0.719 (Table 5).

**Table 4:** Performance comparison of StackER and top-five prediction models in identifying active and inactive compounds for ERα.

Evaluation strategy	Method	ACC	BACC	Sp	Sn	MCC	AUC
Cross-validation	SVM-MACCS	0.887	0.883	0.906	0.860	0.768	0.949
	GLM-KRC	0.892	0.886	0.921	0.851	0.778	0.949
	RF-FP4C	0.893	0.889	0.914	0.865	0.780	0.954
	RF-MACCS	0.895	0.891	0.914	0.869	0.784	0.955
	RF-Pubchem	0.895	0.890	0.926	0.854	0.785	0.954
	StackER	0.925	0.922	0.945	0.899	0.847	0.973
Independent test	SVM-MACCS	0.900	0.899	0.904	0.895	0.796	0.959
	GLM-KRC	0.875	0.875	0.873	0.877	0.747	0.946
	RF-FP4C	0.890	0.891	0.886	0.895	0.777	0.958
	RF-MACCS	0.893	0.892	0.895	0.889	0.781	0.962
	RF-Pubchem	0.883	0.878	0.908	0.848	0.759	0.957
	StackER	0.895	0.894	0.900	0.889	0.786	0.973

**Table 5:** Performance comparison of StackER and top-five prediction models in identifying active and inactive compounds for ERβ.

Evaluation strategy	Method	ACC	BACC	Sn	Sp	MCC	AUC
Cross-validation	SVM-MACCS	0.867	0.855	0.910	0.801	0.719	0.921
	RF-AP2DC	0.872	0.861	0.913	0.809	0.730	0.926
	RF-Pubchem	0.873	0.863	0.912	0.814	0.732	0.930
	RF-MACCS	0.876	0.863	0.918	0.809	0.736	0.928
	RF-FP4C	0.884	0.875	0.917	0.832	0.755	0.931
	StackER	0.918	0.916	0.925	0.907	0.829	0.974
Independent test	SVM-MACCS	0.831	0.807	0.919	0.695	0.641	0.881
	RF-AP2DC	0.864	0.845	0.932	0.758	0.712	0.914
	RF-Pubchem	0.835	0.810	0.926	0.695	0.651	0.915
	RF-MACCS	0.856	0.835	0.932	0.737	0.695	0.914
	RF-FP4C	0.860	0.840	0.932	0.747	0.704	0.925
	StackER	0.864	0.849	0.919	0.779	0.712	0.974

**Figure 4:**
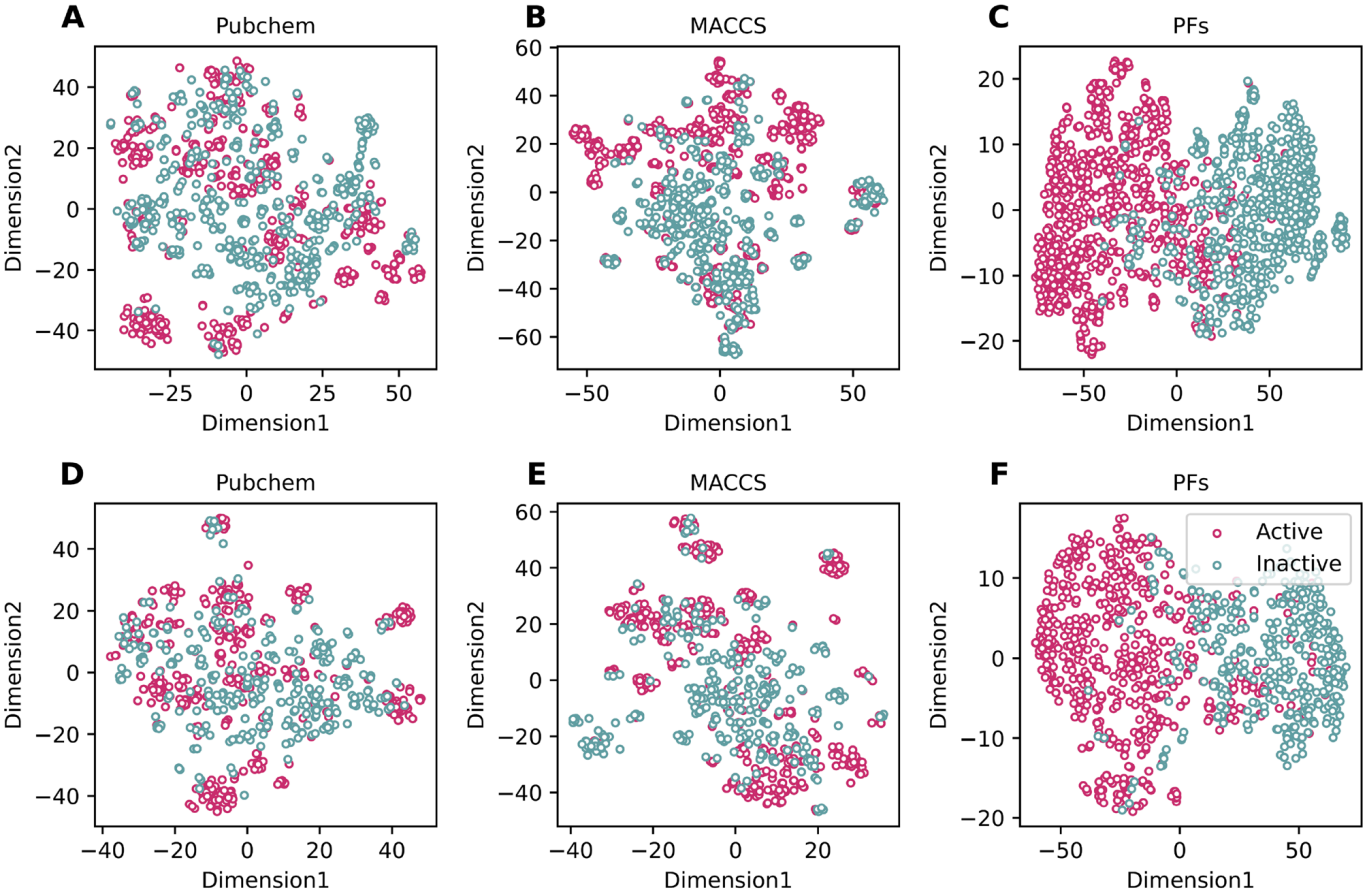
t-SNE distribution of our probabilistic features (PFs) and two informative conventional molecular descriptors for ERα **(A–C)** and ERβ **(D–F)** on the training dataset.

From Fig. 4 and Tables 4 and 5, several points can be observed: (i) StackER achieved a better performance in terms of all six performance metrics over the tenfold cross-validation test for both for ERα and ERβ. Specifically, StackER provided MCC values of 0.847 and 0.829 for ERα and ERβ, which were 6.18–7.95% and 7.41–10.99%, respectively; (ii) As for the independent test results, StackER performed better than almost all of the 5 top-ranked ML classifiers in terms of MCC, with the exception of SVM-MACCS for ERα. However, the performance of StackER was most comparable to that of SVM-MACCS (0.786 versus 0.796) for ERα. In addition, for ERβ, StackER significantly outperformed SVM-MACCS in terms of ACC, BACC, Sp, MCC, and AUC; (iii) StackER attained outstanding AUC values of 0.974 and 0.973 for ERα and ERβ, which were 6.18–7.95% and 7.41–10.99%, respectively; (iv) The PFs were able to create a clearer boundary between the two clusters (i.e., active and inactive) compared to Pubchem and MACCS, demonstrating that the information derived from the PFs is more crucial than conventional molecular descriptors for capturing the distinct patterns between active and inactive samples. Taken together, our comparative results reveal the effectiveness of the stacking strategy to the performance improvement.

### Performance comparison with the existing method

As mentioned above, ERpred^21^ is the only SMILE notation-based approach for predicting ERα and ERβ inhibitors. Since ERpred was not developed based on the up-to-date dataset constructed herein, we implemented ERpred using the same procedure as mentioned in the previous study. Table 6 shows the detailed performance comparison between StackER and ERpred. As can be seen from Table 6**,** for both ERα and ERβ, StackER is superior to ERpred in terms of almost all performance metrics, including ACC, BACC, Sp, MCC, and AUC, on both the training and independent test datasets. In particular, StackER outperformed ERpred as judged by the independent test results, with a 1.75–4.21% increase in Sp, 1.59–0.3. 54% increase in MCC, and 1.33–5.55% increase in AUC, thereby highlighting the effectiveness and robustness of StackER. Furthermore, as StackER attained impressive Sp and MCC values, it could be implied that our proposed model might effectively narrow down the number of candidate drugs against ERα and ERβ.

**Table 6:** Performance comparison of StackER and the existing method on the same training and independent test datasets.

Subtype	Evaluation strategy	Method	ACC	BACC	Sn	Sp	MCC	AUC
ERα	Cross-validation	ERpred	0.897	0.892	0.924	0.860	0.788	0.956
		StackER	0.925	0.922	0.945	0.899	0.847	0.973
	Independent test	ERpred	0.888	0.885	0.900	0.871	0.770	0.960
		StackER	0.895	0.894	0.900	0.889	0.786	0.973
ERβ	Cross-validation	ERpred	0.880	0.870	0.913	0.827	0.746	0.931
		StackER	0.918	0.916	0.925	0.907	0.829	0.974
	Independent test	ERpred	0.848	0.828	0.919	0.737	0.677	0.919
		StackER	0.864	0.849	0.919	0.779	0.712	0.974

### Model interpretation and feature importance analysis

**Figure 5:**
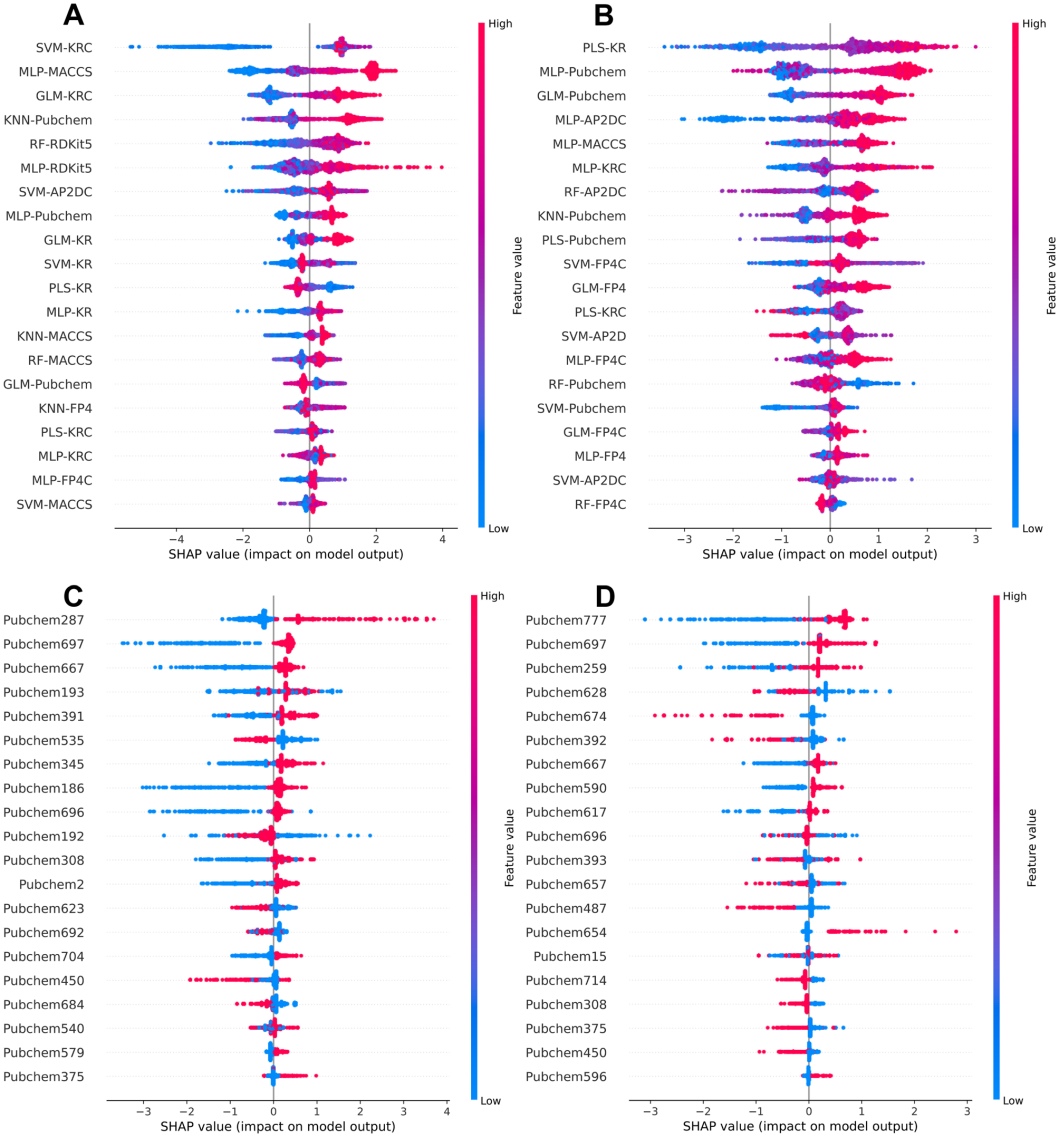
Feature importance analysis based on the SHAP method for StackER **(A**,**B)** and MLP-Pubchem **(C**,**D)**. The impact of each feature on the identification of inhibitors against ERα **(A**,**C)** and ERβ **(A**,**C).** Mean absolute SHAP values, where positive and negatives SHAP values influences the predictions toward positive and negative samples, respectively.

In this section, we utilized the SHAP method^49^ to assess the contribution of each feature on the prediction outputs and identify the most important feature that might be responsible for potential inhibitory effects against ER. Figure 5A, B showcases the top 20 most influential features of StackER for predicting ERα and ERβ inhibitors, respectively, where high and low SHAP values demonstrate that the prediction outputs favour active and inactive classes, respectively. The top-five base-classifiers that were important for predicting ERα and ERβ inhibitors involved (SVM-KR, MLP-MACCS, GLM-KRC, KNN-Pubchem, and RF-RDK5) and (PLS-KR, MLP-Pubchem, GLM-Pubchem, MLP-AP2DC, and MLP-MACCS), respectively. To gain a more profound understanding of the specific features for ERα and ERβ, we also applied the SHAP method to MLP-Pubchem. Figure 5C, D displays the top 20 crucial features for ERα and ERβ, respectively. Furthermore, the particulars of these analyzed substructure fragments, including their general structures and SMARTs patterns, can be found in Table 7.

**Table 7:** Top 10 important features for ERα and ERβ as determined by SHAP method.

Subtype	Rank	Feature	SMARTS pattern	Substructure description	General structure
ERα	1	Pubchem287	C–F	Fluoromethane	
	2	Pubchem697	C–C–C–C–C–C(C)–C	2-methylheptane	
	3	Pubchem667	C=C–C–O–[#1]	Prop-2-en-1-ol	
	4	Pubchem193	>= 3 saturated or aromatic carbon-only ring size 6	Greater than or equal to 3 saturated or aromatic carbon-only six-membered cyclic ring	
	5	Pubchem391	N(~ C)(~ C)(~ C)	*N,N*-dimethylmethanamine	
	6	Pubchem535	O=C–C–C	Propanal	
	7	Pubchem345	C(~ C)(~ H)(~ N)	Ethanamine	
	8	Pubchem186	>= 2 saturated or aromatic carbon-only ring size 6	Greater than or equal to 2 saturated or aromatic carbon-only six-membered cyclic ring	
	9	Pubchem696	C–C–C–C–C–C–C–C	Octane	
	10	Pubchem192	>= 3 any ring size 6	Greater than or equal to 3 six-membered cyclic ring	
ERβ	1	Pubchem777	CC1CCC(O)CC1	4-Methylcyclohexanol	
	2	Pubchem697	C–C–C–C–C–C(C)–C	2-methylheptane	
	3	Pubchem259	>= 3 aromatic rings	Greater than or equal to 3 aromatic carbon-only six-membered cyclic ring	
	4	Pubchem628	C–N–C–C:C	*N*-methylpropan-1-amine	
	5	Pubchem674	N–C–N–C:C	*N*-vinylmethanediamine	
	6	Pubchem392	N(~ C)(~ C)(~ H)	*N*-methylmethanamine	
	7	Pubchem667	C=C–C–O–[#1]	Prop-2-en-1-ol	
	8	Pubchem590	C–C:C–O–[#1]	(*E*)-prop-1-en-1-ol	
	9	Pubchem617	C–C–C–O–[#1]	Propan-1-ol	
	10	Pubchem696	C–C–C–C–C–C–C–C	Octane	

Upon comparing the top 20 important features for ERα and ERβ, we observed that the two subtypes shared five common features, namely Pubchem697, Pubchem667, Pubchem696, Pubchem308, and Pubchem450, which correspond to 2-methylheptane, prop-2-en-1-ol, octane, hydroxyl group, and formimidamide (Table 7). Notably, among these, Pubchem697 and Pubchem667 exhibited a significant impact on both subtypes as ER inhibitors, as indicated by SHAP (Fig. 5C, D). Interestingly, Pubchem697, representing 2-methylheptane, a branched alkane isomeric to octane (i.e., Pubchem696), showed a high SHAP value only for ERα. This feature was also emphasized in our previous work on ERpred^21^, further underscoring its significance. Researchers observed that in derivatives of tamoxifen, a well-known ER inhibitor, the elongated alkyl side chains led to the degradation of ER^50^. In addition, researchers devised a set of diphenylalkane derivatives, incorporating several elongated alkyl chains linked to the hydroxyl group. Subsequently, they assessed the compounds' biological characteristics, encompassing their effects on ER degradation, anti-proliferative properties, transcriptional activity, and binding affinity^51^. Furthermore, in their analysis of the novel compound docking, the scientists observed the interaction between the carboxylic acid of Glu351 in ERα and the hydrogen atom bound to nitrogen. This interaction served as the foundation for the bonding between the ERα hydrophobic groove and the elongated alkyl chain. Consequently, the essential factors contributing to the downregulation of ERα can be attributed to both the nitrogen group and the diphenylheptane with a specific length of extended alkyl chain^52^.

Pubchem667, corresponding to prop-2-en-1-ol, was found to be a potent ER antagonist in a study conducted by Anita et al.^53^. Their research focused on examining the apoptosis in human MCF-7 breast cancer cells and the inhibition of cell proliferation induced by an analogue of Eugenol (4-[4-hydroxy-3-(prop-2-en-1-yl) phenyl]-2-(prop-2-en-1-yl)). Additionally, in the work of Reddy et al.^54^, various compounds containing the prop-2-en-1-ol substructure were tested in vitro, demonstrating their strong efficacy across a broad spectrum of human tumor cell lines, including MCF-7, which is an ER-positive breast cancer cell line. Pubchem308, as shown in Fig. 5C, D and detailed in Table 7, represents a hydroxyl group that gains significance when it is a part of other significant molecular structures, such as bisphenol A (BPA), bisphenol C (BPC), and bisphenol P (BPP). These compounds have been identified as endocrine-disrupting chemicals^55^. The authors of these studies demonstrated that ERα-related transcriptional activity is dependent on the existence of the 4-hydroxyl group in both the A-phenyl and B-phenyl rings of BPA derivatives, which clearly exhibits ER inhibitory effects both in laboratory experiments and in living organisms^56,57^.

Furthermore, nitrogen-containing characteristics, specifically Pubchem391, Pubchem345, and Pubchem375, with high SHAP values, were found to be more prominent in ERα. Conversely, alcohol-containing features, like Pubchem777, Pubchem590, and Pubchem617, had a greater impact on ERβ (Fig. 5C, D and Table 7). The mentioned nitrogen-containing features, associated with *N,N*-dimethylmethanamine, ethanamine, and methanediamine, respectively, serve as precursors for several significant ER inhibitors, including tamoxifen, 4-hydroxy tamoxifen, raloxifene, and their analogues^58–61^. In addition, Makar et al.^62^, highlighted the importance of the *N,N*-dimethylamine side chain in the triphenylethylene-based ER inhibitor tamoxifen. This side chain altered the conformation of helix-12 and inhibited co-activator binding, underscoring its significance in ER inhibition.

The most prominent feature for ERα was identified as fluoromethane (Pubchem287; see Fig. 5C and Table 7), a compound known for its ability to notably enhance various pharmaceutical properties, including potency, metabolic stability, hydrogen bonding, improved binding interactions, and pharmacokinetics, across a range of medications^63^. Furthermore, the inclusion of fluorine atoms in about 20–25% of known drugs highlights the element's importance in medicinal chemistry^64,65^. Scott et al.^66^ conducted a study on the impact of fluorinated analogues on a clinical SERD candidate, and they concluded that the resulting molecules exhibited high quality and advanced profiling stages. Recently, Lu et al.^63^ reported their work on designing and synthesizing fluorinated SERDs based on the clinical drug candidate G1T48 (NCT03455270). Their findings suggested that introducing fluorine atom substitutions into SERDs enhanced overall therapeutic effectiveness, making them superior clinical candidates for orally treating ER-positive breast cancer. As for the top feature for ERβ, Pubchem777 (Fig. 5D and Table 7), which relates to 4-methylcyclohexanol, when oxidized to cyclohexanone and its derivatives, serves as a valuable scaffold in the development of anticancer agents^67^ Such compounds have the potential to act as potent inhibitors of tamoxifen-resistant MCF-7 cancer cells^68,69^. Consequently, the presence of these top features for ERα and ERβ inhibitors underscores the capability of our StackER model to discern the features of significant importance in the field of medicinal chemistry.

### Case study: potential ER inhibitors from FDA-approved drugs

In this section, we applied our StackER model to identify promising ERα inhibitors among existing approved drugs, seeking to maximize therapeutic benefits while minimizing the risks of toxicity. We obtained the data from the DrugBank and applied various filtering criteria, as outlined in the “Materials and methods” section. Following this filtering process, we had a total of 1,737 compounds available for predicting their potential as ER inhibitors using our StackER model. In this context, our primary focus was on identifying potential inhibitors for ERα, as the role of ERβ in breast cancer is intricate and subject to ongoing debate. The results of our predictions for the top 15 potential ERα inhibitors, as determined by our developed model, are presented in Table 8. Notably, among these top 15 compounds, six are directly associated with ERα treatment, including SERMs, SERDs, or substrates of BC resistance proteins. The remaining eight compounds consist of diverse medications, such as antidepressants, antihistamines, anti-cancer agents, and anti-COVID agents.

**Table 8:** Probability, docking scores, and description of 15 selected FDA-approved drugs against ERα as deduced from our StackER model.

DrugBank ID	Compound name	Probability	Docking score (kcal/mol)	Description
DB00481	Raloxifene	0.7577	−10	Non-steriodal SERM
DB06249	Arzoxifene	0.7519	−9.9	SERM
DB08827	Lomitapide	0.7305	−11.2	Cholesterol-lowering drug
DB04841	Flunarizine	0.7188	−10	Selective calcium channel blocker and anti-histamine activity
DB15982	Berotralstat	0.7008	−10.2	Plasma kallikrein inhibitor
DB06401	Bazedoxifene	0.6935	−10.8	Non-steroidal indole-based SERM
DB13292	Pimethixene	0.6929	−7.6	Dopamine antagonist
DB06202	Lasofoxifene	0.6902	−11.6	Non-steroidal SERM
DB16691	Nirmatrelvir	0.6874	−7.6	Anti-covid drug
DB01624	Zuclopenthixol	0.6858	−8.6	Anti-psychotic drug
DB06603	Panobinostat	0.6853	−9	Chemotherapy drug
DB00947	Fulvestrant	0.6801	−9.9	SERD
DB12332	Rucaparib	0.6773	−9.4	PARP inhibitor
DB00434	Cyproheptadine	0.6740	−8.9	Anti-histamine
DB09167	Dosulepin	0.6671	−7.8	Anti-depressant

**Figure 6:**
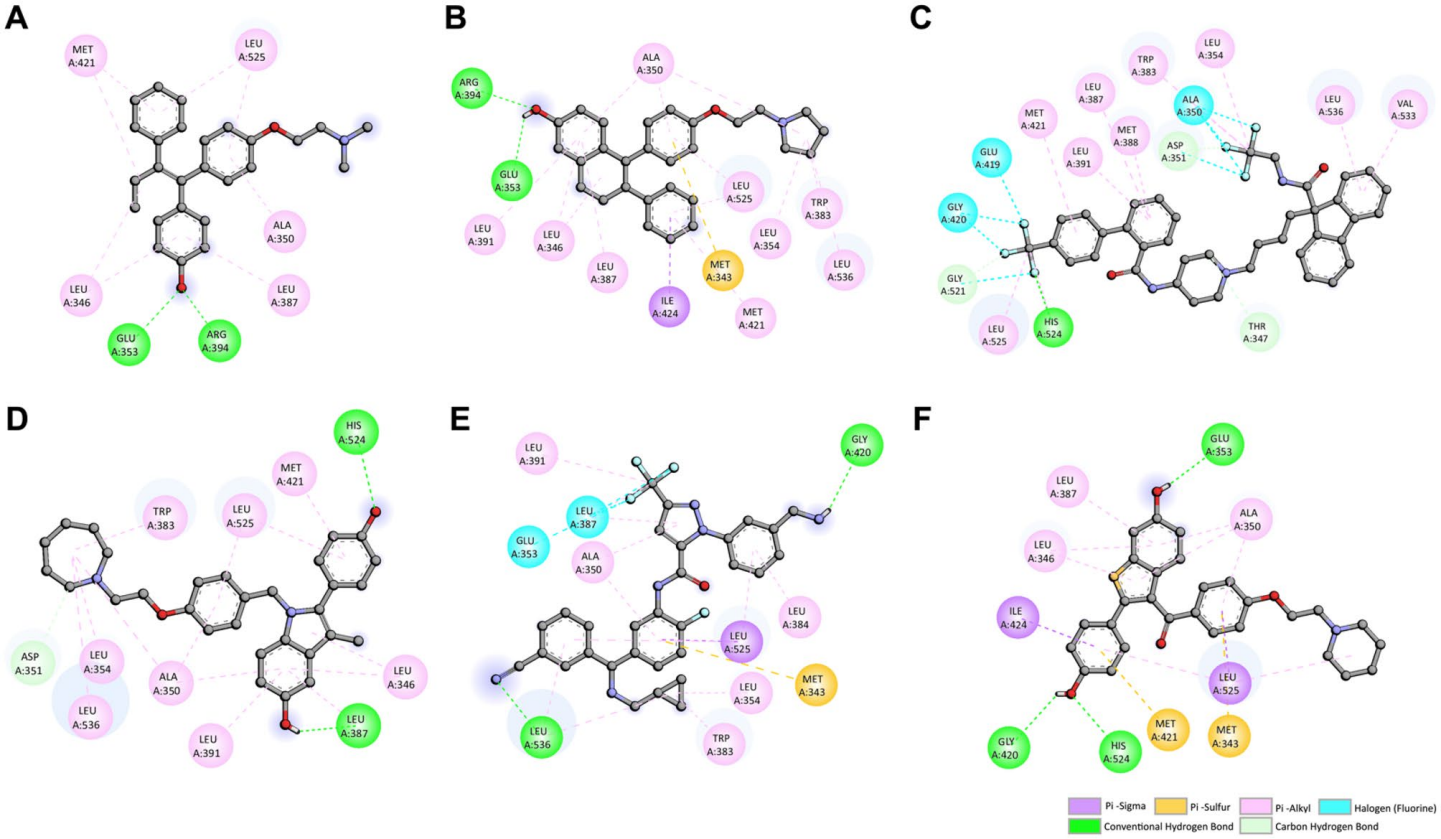
Binding interactions of ERα with OHT **(A)** and the top 5 FDA-approved drugs—Lasofoxifene **(B)**, Lomitapide **(C)**, Bazedoxifene **(D)**, Berotralstat **(E)**, and Raloxifene **(F)**. Residues forming hydrogen bonds are represented in dark green and light green colors while residues forming pi-sigma, pi-alkyl, pi-sulfur and halogen interactions are depicted in purple, pink, orange and blue colors, respectively.

With this in mind, we conducted docking simulations for all of the top compounds using Autodock Vina with the default parameters. The five compounds with the highest docking scores were identified, and their interactions with ERα were further investigated (as shown in the Fig. 6 and Table 8), comparing them to the co-crystal ligand, tamoxifen (OHT). Tamoxifen is a widely recognized SERM used for breast cancer treatment^70,71^ with a long list of side-effects^72^. It forms hydrogen bonds (H-bonds) with Glu353 and Arg394 at distances of 3 Å and 1.98 Å, respectively (as depicted in Fig. 6A). In a similar manner, the top-docked compound, lasofoxifene, with a docking score of −11.6 kcal/mol, is a non-steroidal SERM^73^ and also establishes H-bonds with Glu353 and Arg394 at distances of 2.05 Å and 2.02 Å, respectively (illustrated in Fig. 6B).

It's worth noting that the docking score for OHT was −9.6 kcal/mol. However, OHT did not rank among the top 15 of the predicted potential compounds. This discrepancy may be attributed to the fact that OHT was discovered a long time ago, and our model is trained on the latest data, which includes newer generations of more potent SERMs. In addition, Lainé et al.^73^, recently discovered that lasofoxifene has the potential to treat mutant types of ER^**+**^ metastatic breast cancer. Additionally, among the top 5 docked candidates, three are non-steroidal SERMs (i.e., lasofoxifene, bazedoxifene, and raloxifene). The remaining two, lomitapide and berotralstat, are a lipid-lowering drug and a kallikrein inhibitor, respectively. These two compounds could be strong candidates for drug repurposing.

Lomitapide, initially developed for the treatment of a rare genetic disorder known as familial hypercholesterolemia^74^, achieved a docking score of −11.2 kcal/mol. Figure 6C illustrates the docking interactions of lomitapide with ERα. Notably, lomitapide features two trifluoromethyl groups linked to a nitrogen atom at one end and a carbon atom at the other end, establishing interactions with Ala350, Asp351, Glu419, Glu420, and Gly521, respectively. The substitution of fluorine has been extensively explored in drug design and development to enhance biological activity, metabolic or chemical reactivity, and metabolic or chemical stability^75^. This is primarily attributed to the properties exhibited by fluorine, including lipophilicity, electronegativity, electrostatic interactions, and size^76^. Additionally, lomitapide forms one conventional H-bond with His524 and two carbon-based H-bonds with Asp351 and Gly521. In the work by Zuo et al.^77^, the anti-cancer effects of lomitapide were observed in colorectal cancer. Similarly, in a study by Lee et al.^78^, the authors revealed that lomitapide induces autophagy-dependent cell death in HCT116 colorectal cancer cells. More recently, Wang et al.^79^, demonstrated that lomitapide has the ability to inhibit a key enzyme responsible for the downstream proliferation of pancreatic cancer cells. Moreover, the impressive anti-tumor properties of lomitapide were demonstrated in triple-negative breast cancer (TNBC) cell lines, where researchers observed substantial induction of apoptosis, diminished capacity of TNBC cells to form spheres and colonies while also hindering cell cycle progression^80^.

In contrast, berotralstat has received approval for its use in hereditary angioedema, a rare genetic disorder characterized by recurrent, unpredictable episodes of swelling that affect subcutaneous or submucosal tissues. Berotralstat is an orally administered synthetic small-molecule inhibitor targeting a serine protease called plasma kallikrein. The stimulation of plasma kallikrein leads to the plasma kallikrein/kinin system activation and enhancement. This activation plays a role in the classical complement cascade pathway, the alternative complement pathway, and blood coagulation^81–84^. Nevertheless, no previous reports have documented the anti-cancer properties of berotralstat. In our study, berotralstat emerged as one of the top 5 candidates in both the prediction and docking studies for its potential as an ERα inhibitor, boasting a probability score of 0.7008 and a docking score of -10.2 kcal/mol (as shown in Table 8). Figure 6E displays the interacting residues of berotralstat with ERα. It is notable that berotralstat forms two conventional H-bonds with Gly420 and Leu536, while also establishing pi-sigma and pi-sulfur interactions with Leu525 and Met343, respectively. Furthermore, akin to lomitapide, berotralstat contains a trifluoromethyl group, which interacts with Glu353 and Leu387. This further underscores the significance of fluorine as a sidechain substitution.

It's worth mentioning that the most crucial feature for ERα, as determined by our StackER model and SHAP analysis (as mentioned in the previous section and shown in Fig. 5C and Table 7), was Pubchem287, corresponding to fluoromethane. Consequently, these findings shed light on the potential repurposing of lomitapide and berotralstat as novel therapeutic options for the treatment of ERα-induced cancers.

## Conclusions

In this study, a novel SMILES-based stacked ensemble learning approach, terms StackER, is developed for the accelerated and accurate identification of inhibitors against ERα and ERβ. First, we collected an up-to-date dataset from the ChEMBL database to develop an efficient and effective prediction model. Second, we trained and evaluated several ML classifiers trained with eight ML algorithms combined with nine molecular descriptors. Finally, an optimized stacked approach was constructed based on the combination of selected ML classifiers derived from the two-step feature selection method. Experimental results based on the cross-validation and independent tests, highlighted the effectiveness and robustness of StackER by outperforming the existing method (i.e., ERpred) and several conventional ML classifiers. Three important factors can be attributed to the performance improvement of our developed model: (i) StackER is optimized based on the up-to-date dataset having a larger sample size; (ii) StackER takes advantage of several state-of-the-art ML algorithms and molecular descriptors; and (iii) StackER is developed using the ensemble learning strategy along with the two-step feature selection method. We anticipate that StackER will provide useful insights for the accelerated and large-scale discovery of high potential breast cancer drugs and inspire follow-up research in the future. Although StackER has attained superior predictive performance in comparison to several conventional ML classifiers and the existing method, it still has a few shortcomings, which can be addressed in follow-up works. The first point pertains to developing a two-layer prediction framework that is capable of identifying ERα and ERβ inhibitors (actives or inactives) as well as the inhibitory activity against ERα and ERβ (IC50 bioactivity). The second point is to utilize efficient molecular representation learning (MRL), such as Mol2vec^85^, geometry-enhanced MRL^86^ and self-supervised pretrained learning^87^ strategies. The last point pertains to incorporating StackER with novel ML frameworks, such as a pre-trained language model^88^ and DL-based framework^25,89^.

### Supplementary Information

Supplementary Information.

## Data Availability

The datasets and R source code are available at https://github.com/Shoombuatong/StackER.

## References

[1] W. H. Organization.. *Breast Cancer*. https://www.who.int/news-room/fact-sheets/detail/breast-cancer. Accessed 20 Aug (2023).

[2] Michmerhuizen AR (2022). Estrogen receptor inhibition mediates radiosensitization of ER-positive breast cancer models. NPJ Breast Cancer.

[3] Chen YC (2022). Latest generation estrogen receptor degraders for the treatment of hormone receptor-positive breast cancer. Exp. Opin. Invest. Drugs.

[4] Belachew EB, Sewasew DT (2021). Molecular mechanisms of endocrine resistance in estrogen-positive breast cancer. Front. Endocrinol. (Lausanne).

[5] Zhou Y, Liu X (2020). The role of estrogen receptor beta in breast cancer. Biomark. Res..

[6] Elebro K (2017). High estrogen receptor beta expression is prognostic among adjuvant chemotherapy-treated patients-results from a population-based breast cancer cohort. Clin. Cancer Res..

[7] Patel HK, Bihani T (2018). Selective estrogen receptor modulators (SERMs) and selective estrogen receptor degraders (SERDs) in cancer treatment. Pharmacol. Ther..

[8] Lei JT, Anurag M, Haricharan S, Gou X, Ellis MJ (2019). Endocrine therapy resistance: New insights. Breast.

[9] Robinson DR (2013). Activating ESR1 mutations in hormone-resistant metastatic breast cancer. Nat. Genet..

[10] Mihovic N (2021). Human estrogen receptor alpha antagonists. Part 1: 3-D QSAR-driven rational design of innovative coumarin-related antiestrogens as breast cancer suppressants through structure-based and ligand-based studies. J. Chem. Inf. Model.

[11] Tan H (2020). Structures of endocrine-disrupting chemicals determine binding to and activation of the estrogen receptor alpha and androgen receptor. Environ. Sci. Technol..

[12] Sellami A, Montes M, Lagarde N (2021). Predicting potential endocrine disrupting chemicals binding to estrogen receptor alpha (ERalpha) using a pipeline combining structure-based and ligand-based in silico methods. Int. J. Mol. Sci..

[13] Santaliz-Casiano A (2023). Identification of metabolic pathways contributing to ER(+) breast cancer disparities using a machine-learning pipeline. Sci. Rep..

[14] Bafna D, Ban F, Rennie PS, Singh K, Cherkasov A (2020). Computer-aided ligand discovery for estrogen receptor alpha. Int. J. Mol. Sci..

[15] Zorn KM (2020). Machine learning models for estrogen receptor bioactivity and endocrine disruption prediction. Environ. Sci. Technol..

[16] Pusparini RT, Krisnadhi AA, Firdayani F (2023). MATH: A deep learning approach in QSAR for estrogen receptor alpha inhibitors. Molecules.

[17] Kikiowo B, Ogunleye AJ, Iwaloye O, Ijatuyi TT, Adelakun NS, Alashe WO (2021). Induced fit docking and automated QSAR studies reveal the ER-alpha inhibitory activity of *Cannabis sativa* in breast cancer. Recent Patents Anticancer Drug Discov..

[18] Arvindekar SA (2023). Molecular docking, QSAR, pharmacophore modeling, and dynamics studies of some chromone derivatives for the discovery of anti-breast cancer agents against hormone-dependent breast cancer. J. Biomol. Struct. Dyn..

[19] Laskar YB, Mazumder PB, Talukdar AD (2023). *Hibiscus sabdariffa* anthocyanins are potential modulators of estrogen receptor alpha activity with favourable toxicology: A computational analysis using molecular docking, ADME/Tox prediction, 2D/3D QSAR and molecular dynamics simulation. J. Biomol. Struct. Dyn..

[20] Mendez-Alvarez D, Torres-Rojas MF, Lara-Ramirez EE, Marchat LA, Rivera G (2023). Ligand-based virtual screening, molecular docking, and molecular dynamic simulations of new beta-estrogen receptor activators with potential for pharmacological obesity treatment. Molecules.

[21] Schaduangrat N, Malik AA, Nantasenamat C (2021). ERpred: A web server for the prediction of subtype-specific estrogen receptor antagonists. PeerJ.

[22] Mendez D (2019). ChEMBL: Towards direct deposition of bioassay data. Nucleic Acids Res..

[23] R. C. Team. *R: A Language and Environment for Statistical Computing.* 4.3.0 ed. (R Foundation for Statistical Computing, 2021).

[24] Malik AA, Chotpatiwetchkul W, Phanus-Umporn C, Nantasenamat C, Charoenkwan P, Shoombuatong W (2021). StackHCV: A web-based integrative machine-learning framework for large-scale identification of hepatitis C virus NS5B inhibitors. J. Comput.-Aided Mol. Des..

[25] Schaduangrat N, Anuwongcharoen N, Charoenkwan P, Shoombuatong W (2023). DeepAR: A novel deep learning-based hybrid framework for the interpretable prediction of androgen receptor antagonists. J. Cheminform..

[26] Schaduangrat N, Anuwongcharoen N, Moni MA, Lio P, Charoenkwan P, Shoombuatong W (2022). StackPR is a new computational approach for large-scale identification of progesterone receptor antagonists using the stacking strategy. Sci. Rep..

[27] Yap CW (2011). PaDEL-descriptor: An open source software to calculate molecular descriptors and fingerprints. J. Comput. Chem..

[28] Yu T (2023). Exploring the chemical space of CYP17A1 inhibitors using cheminformatics and machine learning. Molecules.

[29] Yu T, Nantasenamat C, Kachenton S, Anuwongcharoen N, Piacham T (2023). Cheminformatic analysis and machine learning modeling to investigate androgen receptor antagonists to combat prostate cancer. ACS Omega.

[30] Yu, T., Nantasenamat, C., Anuwongcharoen, N. & Piacham, T. Machine learning approaches to investigate the structure–activity relationship of angiotensin-converting enzyme inhibitors. *ACS Omega* (2023). 10.1021/acsomega.3c03225 PMC10666249 38027387

[31] Carhart RE, Smith DH, Venkataraghavan R (1985). Atom pairs as molecular features in structure–activity studies: Definition and applications. J. Chem. Inf. Comput. Sci..

[32] Klekota J, Roth FP (2008). Chemical substructures that enrich for biological activity. Bioinformatics.

[33] RDKit. *Getting Started with the RDKit in Python* [handbook]. https://www.rdkit.org/docs/GettingStartedInPython.html#rdkit-fingerprints (2023).

[34] Kim S (2016). PubChem substance and compound databases. Nucleic Acids Res..

[35] Durant JL, Leland BA, Henry DR, Nourse JG (2002). Reoptimization of MDL keys for use in drug discovery. J. Chem. Inf. Comput. Sci..

[36] Laggner, C. *SMARTS Patterns for Functional Group Classification* (2005).

[37] Sanner MF (1999). Python: A programming language for software integration and development. J. Mol. Graph Model..

[38] R. D. C. Team. *R: A Language and Environment for Statistical Computing* (2010).

[39] Hongjaisee S, Nantasenamat C, Carraway TS, Shoombuatong W (2019). HIVCoR: A sequence-based tool for predicting HIV-1 CRF01_AE coreceptor usage. Comput. Biol. Chem..

[40] Suvannang N (2018). Probing the origin of estrogen receptor alpha inhibition via large-scale QSAR study. RSC Adv..

[41] Charoenkwan P (2022). AMYPred-FRL is a novel approach for accurate prediction of amyloid proteins by using feature representation learning. Sci. Rep..

[42] Ahmad S (2022). SCORPION is a stacking-based ensemble learning framework for accurate prediction of phage virion proteins. Sci. Rep..

[43] Charoenkwan P, Schaduangrat N, Moni MA, Manavalan B, Shoombuatong W (2022). SAPPHIRE: A stacking-based ensemble learning framework for accurate prediction of thermophilic proteins. Comput. Biol. Med..

[44] Johansson MU, Zoete V, Michielin O, Guex N (2012). Defining and searching for structural motifs using DeepView/Swiss-PdbViewer. BMC Bioinform..

[45] Eberhardt J, Santos-Martins D, Tillack AF, Forli S (2021). AutoDock Vina 1.2.0: New docking methods, expanded force field, and Python bindings. J. Chem. Inf. Model.

[46] Kitchen DB, Decornez H, Furr JR, Bajorath J (2004). Docking and scoring in virtual screening for drug discovery: Methods and applications. Nat. Rev. Drug Discov..

[47] Moal IH, Torchala M, Bates PA, Fernandez-Recio J (2013). The scoring of poses in protein-protein docking: Current capabilities and future directions. BMC Bioinform..

[48] Liu H, Papa E, Gramatica P (2008). Evaluation and QSAR modeling on multiple endpoints of estrogen activity based on different bioassays. Chemosphere.

[49] Trevisan, V. *Using SHAP Values to Explain How Your Machine Learning Model Works*. Vol. 2023 (Towards Data Science, 2022).

[50] Shoda T (2015). Synthesis and evaluation of tamoxifen derivatives with a long alkyl side chain as selective estrogen receptor down-regulators. Bioorg Med. Chem..

[51] Misawa T (2017). Design and synthesis of novel selective estrogen receptor degradation inducers based on the diphenylheptane skeleton. Medchemcomm.

[52] Nanjyo S, Ohgane K, Yoshioka H, Makishima M, Hashimoto Y, Noguchi-Yachide T (2019). Structure–activity relationship study of estrogen receptor down-regulators with a diphenylmethane skeleton. Bioorg. Med. Chem..

[53] Anita Y, Radifar M, Kardono LB, Hanafi M, Istyastono EP (2012). Structure-based design of eugenol analogs as potential estrogen receptor antagonists. Bioinformation.

[54] Reddy MV (2012). (*Z*)-1-aryl-3-arylamino-2-propen-1-ones, highly active stimulators of tubulin polymerization: synthesis, structure-activity relationship (SAR), tubulin polymerization, and cell growth inhibition studies. J. Med. Chem..

[55] Matsushima A, Liu X, Okada H, Shimohigashi M, Shimohigashi Y (2010). Bisphenol AF is a full agonist for the estrogen receptor ERalpha but a highly specific antagonist for ERbeta. Environ. Health Perspect..

[56] Zhang Z (2017). Fluorene-9-bisphenol is anti-oestrogenic and may cause adverse pregnancy outcomes in mice. Nat. Commun..

[57] Masuya T, Iwamoto M, Liu X, Matsushima A (2019). Discovery of novel oestrogen receptor alpha agonists and antagonists by screening a revisited privileged structure moiety for nuclear receptors. Sci. Rep..

[58] Ohta K, Chiba Y, Kaise A, Endo Y (2015). Structure-activity relationship study of diphenylamine-based estrogen receptor (ER) antagonists. Bioorg. Med. Chem..

[59] Sharma D, Kumar S, Narasimhan B (2018). Estrogen alpha receptor antagonists for the treatment of breast cancer: A review. Chem. Center J..

[60] Ohta K, Chiba Y, Ogawa T, Endo Y (2008). Promising core structure for nuclear receptor ligands: Design and synthesis of novel estrogen receptor ligands based on diphenylamine skeleton. Bioorg. Med. Chem. Lett..

[61] Guo WY, Zeng SM, Deora GS, Li QS, Ruan BF (2019). Estrogen receptor alpha (ERalpha)-targeting compounds and derivatives: Recent advances in structural modification and bioactivity. Curr. Top. Med. Chem..

[62] Makar S, Saha T, Swetha R, Gutti G, Kumar A, Singh SK (2020). Rational approaches of drug design for the development of selective estrogen receptor modulators (SERMs), implicated in breast cancer. Bioorg. Chem..

[63] Lu Y (2023). Design, synthesis and biological evaluation of fluorinated selective estrogen receptor degraders (FSERDs)—A promising strategy for advanced ER positive breast cancer. Eur. J. Med. Chem..

[64] Bohm HJ (2004). Fluorine in medicinal chemistry. Chembiochem.

[65] Muller K, Faeh C, Diederich F (2007). Fluorine in pharmaceuticals: Looking beyond intuition. Science.

[66] Scott JS (2020). Addition of fluorine and a late-stage functionalization (LSF) of the oral SERD AZD9833. ACS Med. Chem. Lett..

[67] Al-Majid AM (2019). Synthesis of pyridine-dicarboxamide-cyclohexanone derivatives: Anticancer and alpha-glucosidase inhibitory activities and in silico study. Molecules.

[68] Leung E, Rewcastle GW, Joseph WR, Rosengren RJ, Larsen L, Baguley BC (2012). Identification of cyclohexanone derivatives that act as catalytic inhibitors of topoisomerase I: Effects on tamoxifen-resistant MCF-7 cancer cells. Invest. New Drugs.

[69] Yeap SK (2021). Induction of apoptosis and regulation of microRNA expression by (2*E*,6*E*)-2,6-bis-(4-hydroxy-3-methoxybenzylidene)-cyclohexanone (BHMC) treatment on MCF-7 breast cancer cells. Molecules.

[70] Marina D, Rasmussen AK, Buch-Larsen K, Gillberg L, Andersson M, Schwarz P (2023). Influence of the anti-oestrogens tamoxifen and letrozole on thyroid function in women with early and advanced breast cancer: A systematic review. Cancer Med..

[71] Ghanavati M (2023). Tamoxifen use and risk of endometrial cancer in breast cancer patients: A systematic review and dose-response meta-analysis. Cancer Rep. (Hoboken).

[72] Farrar, M. C. & Jacobs, T. F. *Tamoxifen*. (StatPearlsTreasure Island, 2023).

[73] Laine M (2021). Lasofoxifene as a potential treatment for therapy-resistant ER-positive metastatic breast cancer. Breast Cancer Res..

[74] Ajufo E, Rader DJ (2018). New therapeutic approaches for familial hypercholesterolemia. Annu. Rev. Med..

[75] Kirk KL (2006). Selective fluorination in drug design and development: An overview of biochemical rationales. Curr. Top. Med. Chem..

[76] Hagmann WK (2008). The many roles for fluorine in medicinal chemistry. J. Med. Chem..

[77] Zuo Q (2021). Targeting PP2A with lomitapide suppresses colorectal tumorigenesis through the activation of AMPK/Beclin1-mediated autophagy. Cancer Lett..

[78] Lee B (2022). Lomitapide, a cholesterol-lowering drug, is an anticancer agent that induces autophagic cell death via inhibiting mTOR. Cell Death Dis..

[79] Wang Y (2023). Repositioning Lomitapide to block ZDHHC5-dependant palmitoylation on SSTR5 leads to anti-proliferation effect in preclinical pancreatic cancer models. Cell Death Discov..

[80] Sen P, Kandasamy T, Ghosh SS (2023). Multi-targeting TACE/ADAM17 and gamma-secretase of notch signalling pathway in TNBC via drug repurposing approach using Lomitapide. Cell Signal.

[81] Farkas H, Balla Z (2023). A review of berotralstat for the treatment of hereditary angioedema. Expert Rev. Clin. Immunol..

[82] Busse P, Kaplan A (2022). Specific targeting of plasma kallikrein for treatment of hereditary angioedema: A revolutionary decade. J. Allergy Clin. Immunol. Pract..

[83] Kaplan AP, Joseph K (2017). Pathogenesis of hereditary angioedema: The role of the Bradykinin-forming cascade. Immunol. Allergy Clin. N. Am..

[84] Hwang JR, Hwang G, Johri A, Craig T (2019). Oral plasma kallikrein inhibitor BCX7353 for treatment of hereditary angioedema. Immunotherapy.

[85] Jaeger S, Fulle S, Turk S (2018). Mol2vec: Unsupervised machine learning approach with chemical intuition. J. Chem. Inf. Model..

[86] Fang X (2022). Geometry-enhanced molecular representation learning for property prediction. Nat. Mach. Intell..

[87] Zeng X (2022). Accurate prediction of molecular properties and drug targets using a self-supervised image representation learning framework. Nat. Mach. Intell..

[88] Li Z, Jin J, Long W, Wei L (2023). PLPMpro: Enhancing promoter sequence prediction with prompt-learning based pre-trained language model. Comput. Biol. Med..

[89] Xie R (2021). DeepVF: A deep learning-based hybrid framework for identifying virulence factors using the stacking strategy. Brief. Bioinform..

