# Supplementary material for: StackER: a novel SMILES-based stacked approach for the accelerated and efficient discovery of ERα and ERβ antagonists
Source: Sci Rep. 2023 Dec 27;13:22994. doi: 10.1038/s41598-023-50393-w (PMC10752908; doi:10.1038/s41598-023-50393-w)
Supplement: Supplementary file 1 — Supplementary Information. [file 41598_2023_50393_MOESM1_ESM.docx]

### **Evaluation criteria**

To assess the predictive capability of our proposed model, we employed six well-known metrics, including Matthew’s coefficient correlation (MCC), area under the receiver operating characteristic (ROC) curve (AUC), accuracy (ACC), balanced accuracy (BACC), specificity (Sp), and sensitivity (Sn). These metrics are described as follows:

| $Sn=\frac{TP}{\left( TP+FN \right)}$ | (1) |
| --- | --- |
| $Sp=\frac{TN}{\left( TN+FP \right)}$ | (2) |
| $MCC=\frac{TP\times TN-FP\times FN}{\sqrt{(TP+FP)(TP+FN)(TN+FP)(TN+FN)}}$ | (3) |
| $ACC=\frac{TP+TN}{\left( TP+TN+FP+FN \right)}$ | (4) |
| $BACC=\frac{Sn+Sp}{2}$ | (5) |

where TP, FP, TN, and FN the number of true positive, false positive, true negative, false positive and false negative compounds, respectively.

## **Supplementary Figures**


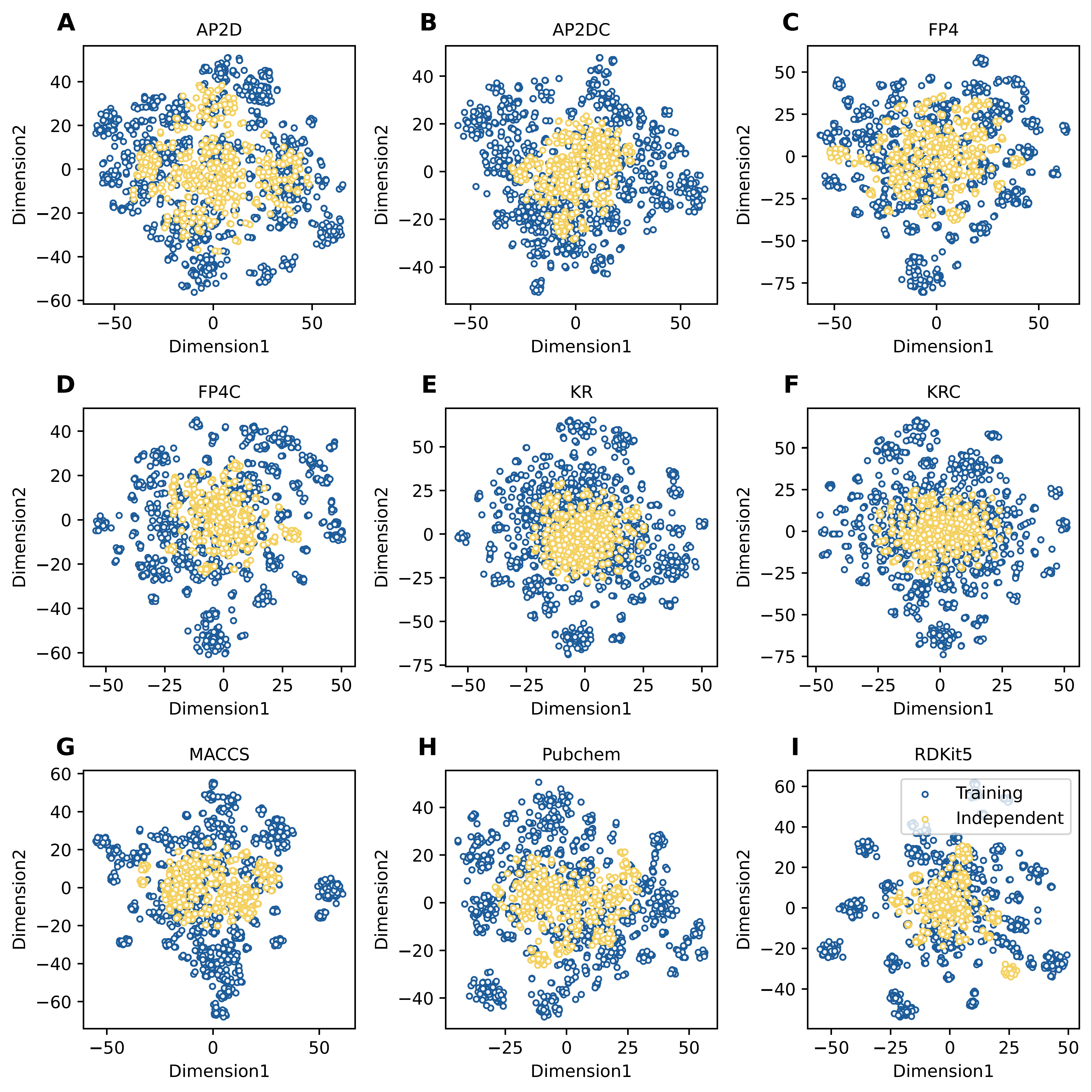


**Figure S1.** t-SNE distribution of nine conventional molecular descriptors for ERα on the training dataset.


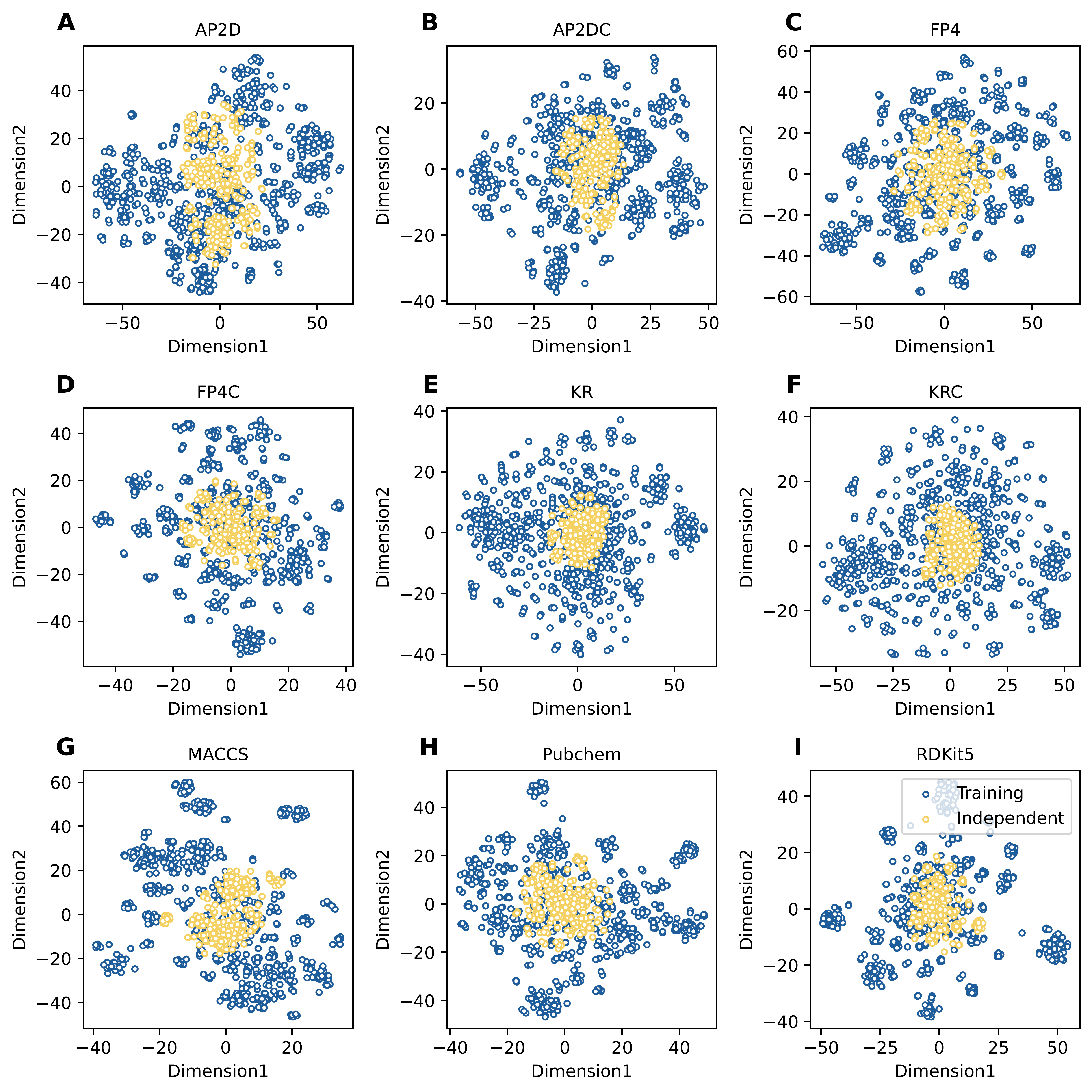


**Figure S2.** t-SNE distribution of nine conventional molecular descriptors for ERβ on the training dataset

## **Supplementary Tables**

**Table S1.** Information of parameter settings for eight ML methods used in this study.

| **Method** | **Parameters** | **Search space** |
| --- | --- | --- |
| PLS | *ncomp* | [1, 2, 3, 4, 5] |
| KNN | *k* | [1, 3, 5, 7, 9] |
| GLM | *alpha* | 1 |
|  | *lambda* | [0.0001, 0.001, 0.01] |
| rpart | *cp* | [0.021, 0.024, 0.051, 0.053, 0.250] |
| MLP | *size* | [1, 3, 5, 7, 9] |
| SVM | *C* | [0.25, 0.5, 1, 2, 4] |
| RF | *mtry* | [5, 7, 10, 15, 20, 25] |
|  | *ntree* | [20, 50, 100, 200, 300] |
| XGB | *nrounds* | [20, 50, 100, 200, 300] |
|  | *max_depth* | [3, 5, 7, 9, 10] |
|  | *eta* | [0.1, 0.2, 0.3] |
|  | *gamma* | 0 |
|  | *colsample_bytree* | 0.7 |
|  | *min_child_weight* | 5 |
|  | *subsample* | 0.5 |

**Table S2** Cross-validation results of 72 base-classifiers developed using eight different ML algorithms and nine molecular descriptors for ERα.

| **Descriptor** | **Method** | **ACC** | **BACC** | **Sn** | **Sp** | **MCC** | **AUC** |
| --- | --- | --- | --- | --- | --- | --- | --- |
| AP2D | GLM | 0.803 | 0.796 | 0.840 | 0.753 | 0.595 | 0.891 |
|  | KNN | 0.804 | 0.796 | 0.852 | 0.740 | 0.597 | 0.939 |
|  | MLP | 0.816 | 0.815 | 0.826 | 0.803 | 0.627 | 0.896 |
|  | PLS | 0.806 | 0.799 | 0.848 | 0.750 | 0.602 | 0.888 |
|  | RF | 0.833 | 0.829 | 0.855 | 0.803 | 0.658 | 0.909 |
|  | rpart | 0.781 | 0.784 | 0.764 | 0.804 | 0.563 | 0.903 |
|  | SVM | 0.824 | 0.823 | 0.832 | 0.813 | 0.642 | 0.880 |
|  | XGB | 0.845 | 0.841 | 0.867 | 0.815 | 0.682 | 0.926 |
| AP2DC | GLM | 0.831 | 0.827 | 0.858 | 0.796 | 0.655 | 0.900 |
|  | KNN | 0.847 | 0.840 | 0.888 | 0.793 | 0.686 | 0.972 |
|  | MLP | 0.855 | 0.850 | 0.885 | 0.815 | 0.703 | 0.914 |
|  | PLS | 0.810 | 0.804 | 0.844 | 0.765 | 0.611 | 0.887 |
|  | RF | 0.887 | 0.882 | 0.912 | 0.853 | 0.768 | 0.953 |
|  | rpart | 0.801 | 0.790 | 0.866 | 0.715 | 0.591 | 0.920 |
|  | SVM | 0.834 | 0.834 | 0.836 | 0.831 | 0.663 | 0.904 |
|  | XGB | 0.875 | 0.872 | 0.896 | 0.847 | 0.745 | 0.947 |
| FP4 | GLM | 0.841 | 0.836 | 0.871 | 0.800 | 0.674 | 0.908 |
|  | KNN | 0.838 | 0.832 | 0.870 | 0.794 | 0.667 | 0.946 |
|  | MLP | 0.865 | 0.862 | 0.882 | 0.843 | 0.725 | 0.920 |
|  | PLS | 0.842 | 0.835 | 0.881 | 0.790 | 0.676 | 0.907 |
|  | RF | 0.883 | 0.878 | 0.910 | 0.846 | 0.760 | 0.942 |
|  | rpart | 0.795 | 0.787 | 0.841 | 0.734 | 0.579 | 0.898 |
|  | SVM | 0.855 | 0.850 | 0.883 | 0.818 | 0.703 | 0.924 |
|  | XGB | 0.865 | 0.859 | 0.900 | 0.818 | 0.722 | 0.933 |
| FP4C | GLM | 0.850 | 0.847 | 0.867 | 0.826 | 0.693 | 0.919 |
|  | KNN | 0.833 | 0.824 | 0.885 | 0.763 | 0.657 | 0.933 |
|  | MLP | 0.848 | 0.841 | 0.885 | 0.797 | 0.687 | 0.918 |
|  | PLS | 0.840 | 0.836 | 0.859 | 0.813 | 0.672 | 0.914 |
|  | RF | 0.893 | 0.889 | 0.914 | 0.865 | 0.780 | 0.954 |
|  | rpart | 0.799 | 0.788 | 0.867 | 0.709 | 0.587 | 0.896 |
|  | SVM | 0.870 | 0.866 | 0.894 | 0.838 | 0.734 | 0.932 |
|  | XGB | 0.882 | 0.877 | 0.910 | 0.844 | 0.758 | 0.945 |
| KR | GLM | 0.883 | 0.878 | 0.915 | 0.841 | 0.761 | 0.949 |
|  | KNN | 0.815 | 0.803 | 0.879 | 0.728 | 0.618 | 0.955 |
|  | MLP | 0.858 | 0.854 | 0.880 | 0.828 | 0.709 | 0.938 |
|  | PLS | 0.878 | 0.871 | 0.918 | 0.824 | 0.749 | 0.938 |
|  | RF | 0.873 | 0.869 | 0.900 | 0.838 | 0.741 | 0.941 |
|  | rpart | 0.775 | 0.775 | 0.776 | 0.774 | 0.546 | 0.909 |
|  | SVM | 0.859 | 0.859 | 0.859 | 0.859 | 0.714 | 0.927 |
|  | XGB | 0.860 | 0.856 | 0.885 | 0.826 | 0.714 | 0.945 |
| KRC | GLM | 0.892 | 0.886 | 0.921 | 0.851 | 0.778 | 0.949 |
|  | KNN | 0.837 | 0.825 | 0.907 | 0.743 | 0.666 | 0.940 |
|  | MLP | 0.878 | 0.875 | 0.896 | 0.853 | 0.750 | 0.941 |
|  | PLS | 0.882 | 0.876 | 0.918 | 0.834 | 0.758 | 0.941 |
|  | RF | 0.873 | 0.868 | 0.901 | 0.835 | 0.739 | 0.942 |
|  | rpart | 0.780 | 0.778 | 0.790 | 0.766 | 0.554 | 0.903 |
|  | SVM | 0.860 | 0.861 | 0.854 | 0.868 | 0.717 | 0.926 |
|  | XGB | 0.872 | 0.867 | 0.900 | 0.834 | 0.737 | 0.947 |
| MACCS | GLM | 0.855 | 0.849 | 0.889 | 0.810 | 0.703 | 0.935 |
|  | KNN | 0.863 | 0.855 | 0.909 | 0.800 | 0.718 | 0.969 |
|  | MLP | 0.872 | 0.867 | 0.896 | 0.838 | 0.737 | 0.935 |
|  | PLS | 0.861 | 0.853 | 0.906 | 0.800 | 0.714 | 0.932 |
|  | RF | 0.895 | 0.891 | 0.914 | 0.869 | 0.784 | 0.955 |
|  | rpart | 0.789 | 0.770 | 0.898 | 0.641 | 0.567 | 0.874 |
|  | SVM | 0.887 | 0.883 | 0.906 | 0.860 | 0.768 | 0.949 |
|  | XGB | 0.885 | 0.882 | 0.905 | 0.859 | 0.765 | 0.955 |
| Pubchem | GLM | 0.865 | 0.860 | 0.898 | 0.821 | 0.724 | 0.936 |
|  | KNN | 0.848 | 0.840 | 0.896 | 0.784 | 0.688 | 0.962 |
|  | MLP | 0.875 | 0.871 | 0.898 | 0.843 | 0.743 | 0.943 |
|  | PLS | 0.867 | 0.859 | 0.908 | 0.810 | 0.726 | 0.941 |
|  | RF | 0.895 | 0.890 | 0.926 | 0.854 | 0.785 | 0.954 |
|  | rpart | 0.821 | 0.809 | 0.894 | 0.724 | 0.633 | 0.920 |
|  | SVM | 0.883 | 0.880 | 0.905 | 0.854 | 0.761 | 0.939 |
|  | XGB | 0.884 | 0.879 | 0.913 | 0.846 | 0.762 | 0.954 |
| RDK5 | GLM | 0.560 | 0.532 | 0.724 | 0.340 | 0.068 | 0.533 |
|  | KNN | 0.548 | 0.528 | 0.660 | 0.396 | 0.058 | 0.620 |
|  | MLP | 0.539 | 0.520 | 0.653 | 0.387 | 0.041 | 0.546 |
|  | PLS | 0.551 | 0.524 | 0.712 | 0.335 | 0.050 | 0.533 |
|  | RF | 0.563 | 0.529 | 0.762 | 0.296 | 0.065 | 0.547 |
|  | rpart | 0.574 | 0.508 | 0.954 | 0.062 | 0.035 | 0.918 |
|  | SVM | 0.569 | 0.499 | 0.973 | 0.025 | -0.007 | 0.535 |
|  | XGB | 0.549 | 0.517 | 0.731 | 0.303 | 0.038 | 0.538 |

**Table S3** Independent test results of 72 base-classifiers developed using eight different ML algorithms and nine molecular descriptors for ERα.

| **Descriptor** | **Method** | **ACC** | **BACC** | **Sn** | **Sp** | **MCC** | **AUC** |
| --- | --- | --- | --- | --- | --- | --- | --- |
| AP2D | GLM | 0.805 | 0.806 | 0.799 | 0.813 | 0.607 | 0.876 |
|  | KNN | 0.808 | 0.803 | 0.834 | 0.772 | 0.606 | 0.945 |
|  | MLP | 0.833 | 0.829 | 0.852 | 0.807 | 0.658 | 0.888 |
|  | PLS | 0.815 | 0.818 | 0.799 | 0.836 | 0.630 | 0.882 |
|  | RF | 0.850 | 0.856 | 0.817 | 0.895 | 0.704 | 0.915 |
|  | rpart | 0.703 | 0.696 | 0.738 | 0.655 | 0.393 | 0.874 |
|  | SVM | 0.800 | 0.808 | 0.751 | 0.865 | 0.610 | 0.868 |
|  | XGB | 0.863 | 0.865 | 0.847 | 0.883 | 0.724 | 0.933 |
| AP2DC | GLM | 0.818 | 0.819 | 0.808 | 0.830 | 0.633 | 0.890 |
|  | KNN | 0.850 | 0.846 | 0.873 | 0.819 | 0.693 | 0.977 |
|  | MLP | 0.835 | 0.839 | 0.812 | 0.865 | 0.671 | 0.896 |
|  | PLS | 0.820 | 0.818 | 0.830 | 0.807 | 0.634 | 0.879 |
|  | RF | 0.873 | 0.877 | 0.847 | 0.906 | 0.747 | 0.962 |
|  | rpart | 0.808 | 0.802 | 0.843 | 0.760 | 0.605 | 0.935 |
|  | SVM | 0.835 | 0.843 | 0.790 | 0.895 | 0.678 | 0.901 |
|  | XGB | 0.875 | 0.877 | 0.860 | 0.895 | 0.749 | 0.950 |
| FP4 | GLM | 0.823 | 0.820 | 0.838 | 0.801 | 0.638 | 0.898 |
|  | KNN | 0.863 | 0.861 | 0.873 | 0.848 | 0.720 | 0.961 |
|  | MLP | 0.853 | 0.853 | 0.847 | 0.860 | 0.702 | 0.934 |
|  | PLS | 0.823 | 0.820 | 0.838 | 0.801 | 0.638 | 0.892 |
|  | RF | 0.875 | 0.877 | 0.865 | 0.889 | 0.748 | 0.940 |
|  | rpart | 0.788 | 0.780 | 0.830 | 0.731 | 0.564 | 0.903 |
|  | SVM | 0.850 | 0.847 | 0.869 | 0.825 | 0.694 | 0.925 |
|  | XGB | 0.858 | 0.858 | 0.856 | 0.860 | 0.712 | 0.934 |
| FP4C | GLM | 0.855 | 0.856 | 0.852 | 0.860 | 0.707 | 0.928 |
|  | KNN | 0.835 | 0.828 | 0.878 | 0.778 | 0.661 | 0.927 |
|  | MLP | 0.885 | 0.885 | 0.882 | 0.889 | 0.767 | 0.937 |
|  | PLS | 0.838 | 0.838 | 0.834 | 0.842 | 0.672 | 0.909 |
|  | RF | 0.890 | 0.891 | 0.886 | 0.895 | 0.777 | 0.958 |
|  | rpart | 0.803 | 0.788 | 0.886 | 0.690 | 0.594 | 0.907 |
|  | SVM | 0.873 | 0.872 | 0.873 | 0.871 | 0.741 | 0.936 |
|  | XGB | 0.878 | 0.877 | 0.878 | 0.877 | 0.752 | 0.956 |
| KR | GLM | 0.873 | 0.872 | 0.873 | 0.871 | 0.741 | 0.946 |
|  | KNN | 0.838 | 0.825 | 0.908 | 0.743 | 0.667 | 0.975 |
|  | MLP | 0.870 | 0.871 | 0.865 | 0.877 | 0.737 | 0.932 |
|  | PLS | 0.880 | 0.877 | 0.895 | 0.860 | 0.755 | 0.940 |
|  | RF | 0.888 | 0.888 | 0.882 | 0.895 | 0.772 | 0.949 |
|  | rpart | 0.803 | 0.810 | 0.760 | 0.860 | 0.613 | 0.903 |
|  | SVM | 0.855 | 0.859 | 0.834 | 0.883 | 0.711 | 0.925 |
|  | XGB | 0.890 | 0.888 | 0.900 | 0.877 | 0.776 | 0.959 |
| KRC | GLM | 0.875 | 0.875 | 0.873 | 0.877 | 0.747 | 0.946 |
|  | KNN | 0.850 | 0.842 | 0.900 | 0.784 | 0.692 | 0.952 |
|  | MLP | 0.873 | 0.871 | 0.882 | 0.860 | 0.740 | 0.941 |
|  | PLS | 0.880 | 0.877 | 0.900 | 0.854 | 0.755 | 0.929 |
|  | RF | 0.890 | 0.890 | 0.891 | 0.889 | 0.777 | 0.955 |
|  | rpart | 0.795 | 0.799 | 0.769 | 0.830 | 0.593 | 0.901 |
|  | SVM | 0.855 | 0.859 | 0.830 | 0.889 | 0.712 | 0.917 |
|  | XGB | 0.893 | 0.894 | 0.886 | 0.901 | 0.783 | 0.960 |
| MACCS | GLM | 0.840 | 0.836 | 0.865 | 0.807 | 0.673 | 0.928 |
|  | KNN | 0.863 | 0.856 | 0.900 | 0.813 | 0.718 | 0.978 |
|  | MLP | 0.843 | 0.839 | 0.860 | 0.819 | 0.678 | 0.922 |
|  | PLS | 0.838 | 0.834 | 0.860 | 0.807 | 0.668 | 0.930 |
|  | RF | 0.893 | 0.892 | 0.895 | 0.889 | 0.781 | 0.962 |
|  | rpart | 0.768 | 0.754 | 0.847 | 0.661 | 0.521 | 0.894 |
|  | SVM | 0.900 | 0.899 | 0.904 | 0.895 | 0.796 | 0.959 |
|  | XGB | 0.873 | 0.875 | 0.860 | 0.889 | 0.744 | 0.951 |
| Pubchem | GLM | 0.870 | 0.869 | 0.878 | 0.860 | 0.735 | 0.943 |
|  | KNN | 0.865 | 0.857 | 0.913 | 0.801 | 0.723 | 0.979 |
|  | MLP | 0.843 | 0.841 | 0.852 | 0.830 | 0.680 | 0.935 |
|  | PLS | 0.848 | 0.845 | 0.865 | 0.825 | 0.689 | 0.920 |
|  | RF | 0.883 | 0.878 | 0.908 | 0.848 | 0.759 | 0.957 |
|  | rpart | 0.803 | 0.786 | 0.900 | 0.673 | 0.595 | 0.904 |
|  | SVM | 0.870 | 0.871 | 0.865 | 0.877 | 0.737 | 0.939 |
|  | XGB | 0.880 | 0.879 | 0.886 | 0.871 | 0.756 | 0.961 |
| RDK5 | GLM | 0.583 | 0.559 | 0.721 | 0.398 | 0.124 | 0.585 |
|  | KNN | 0.530 | 0.512 | 0.638 | 0.386 | 0.024 | 0.597 |
|  | MLP | 0.523 | 0.533 | 0.459 | 0.608 | 0.067 | 0.582 |
|  | PLS | 0.575 | 0.552 | 0.712 | 0.392 | 0.109 | 0.560 |
|  | RF | 0.578 | 0.544 | 0.773 | 0.316 | 0.099 | 0.573 |
|  | rpart | 0.540 | 0.525 | 0.629 | 0.421 | 0.051 | 0.616 |
|  | SVM | 0.560 | 0.497 | 0.930 | 0.064 | -0.011 | 0.586 |
|  | XGB | 0.555 | 0.536 | 0.668 | 0.404 | 0.074 | 0.543 |

**Table S4** Cross-validation results of 72 base-classifiers developed using eight different ML algorithms and nine molecular descriptors for ERβ.

| **Descriptor** | **Method** | **ACC** | **BACC** | **Sn** | **Sp** | **MCC** | **AUC** |
| --- | --- | --- | --- | --- | --- | --- | --- |
| AP2D | GLM | 0.816 | 0.795 | 0.699 | 0.891 | 0.608 | 0.866 |
|  | KNN | 0.795 | 0.767 | 0.644 | 0.891 | 0.560 | 0.936 |
|  | MLP | 0.817 | 0.802 | 0.734 | 0.871 | 0.613 | 0.862 |
|  | PLS | 0.803 | 0.778 | 0.662 | 0.893 | 0.579 | 0.857 |
|  | RF | 0.832 | 0.809 | 0.705 | 0.913 | 0.642 | 0.892 |
|  | rpart | 0.733 | 0.700 | 0.551 | 0.850 | 0.424 | 0.875 |
|  | SVM | 0.803 | 0.787 | 0.715 | 0.859 | 0.582 | 0.855 |
|  | XGB | 0.831 | 0.815 | 0.745 | 0.886 | 0.641 | 0.887 |
| AP2DC | GLM | 0.829 | 0.809 | 0.721 | 0.898 | 0.636 | 0.874 |
|  | KNN | 0.803 | 0.772 | 0.633 | 0.912 | 0.579 | 0.939 |
|  | MLP | 0.810 | 0.795 | 0.729 | 0.862 | 0.598 | 0.867 |
|  | PLS | 0.813 | 0.790 | 0.684 | 0.896 | 0.601 | 0.854 |
|  | RF | 0.872 | 0.861 | 0.809 | 0.913 | 0.730 | 0.926 |
|  | rpart | 0.775 | 0.743 | 0.598 | 0.888 | 0.517 | 0.890 |
|  | SVM | 0.812 | 0.801 | 0.753 | 0.850 | 0.604 | 0.881 |
|  | XGB | 0.859 | 0.852 | 0.819 | 0.884 | 0.704 | 0.910 |
| FP4 | GLM | 0.816 | 0.791 | 0.676 | 0.906 | 0.608 | 0.877 |
|  | KNN | 0.808 | 0.788 | 0.697 | 0.879 | 0.591 | 0.926 |
|  | MLP | 0.831 | 0.818 | 0.761 | 0.876 | 0.642 | 0.871 |
|  | PLS | 0.800 | 0.768 | 0.622 | 0.913 | 0.572 | 0.857 |
|  | RF | 0.851 | 0.833 | 0.755 | 0.912 | 0.683 | 0.921 |
|  | rpart | 0.779 | 0.749 | 0.614 | 0.884 | 0.526 | 0.895 |
|  | SVM | 0.839 | 0.823 | 0.747 | 0.898 | 0.658 | 0.899 |
|  | XGB | 0.818 | 0.801 | 0.721 | 0.881 | 0.614 | 0.885 |
| FP4C | GLM | 0.829 | 0.810 | 0.723 | 0.896 | 0.636 | 0.872 |
|  | KNN | 0.828 | 0.810 | 0.731 | 0.889 | 0.634 | 0.959 |
|  | MLP | 0.846 | 0.836 | 0.787 | 0.884 | 0.676 | 0.876 |
|  | PLS | 0.816 | 0.786 | 0.649 | 0.923 | 0.609 | 0.861 |
|  | RF | 0.884 | 0.875 | 0.832 | 0.917 | 0.755 | 0.931 |
|  | rpart | 0.766 | 0.730 | 0.566 | 0.893 | 0.496 | 0.883 |
|  | SVM | 0.851 | 0.839 | 0.787 | 0.891 | 0.684 | 0.911 |
|  | XGB | 0.857 | 0.845 | 0.790 | 0.900 | 0.697 | 0.918 |
| KR | GLM | 0.570 | 0.520 | 0.293 | 0.747 | 0.043 | 0.528 |
|  | KNN | 0.558 | 0.514 | 0.311 | 0.716 | 0.029 | 0.603 |
|  | MLP | 0.547 | 0.532 | 0.463 | 0.600 | 0.062 | 0.533 |
|  | PLS | 0.560 | 0.506 | 0.258 | 0.753 | 0.013 | 0.523 |
|  | RF | 0.607 | 0.504 | 0.037 | 0.971 | 0.023 | 0.542 |
|  | rpart | 0.603 | 0.495 | 0.003 | 0.986 | -0.056 | 0.986 |
|  | SVM | 0.607 | 0.499 | 0.008 | 0.990 | -0.011 | 0.507 |
|  | XGB | 0.565 | 0.481 | 0.096 | 0.866 | -0.058 | 0.492 |
| KRC | GLM | 0.547 | 0.498 | 0.274 | 0.721 | -0.005 | 0.500 |
|  | KNN | 0.548 | 0.510 | 0.340 | 0.680 | 0.022 | 0.611 |
|  | MLP | 0.523 | 0.508 | 0.441 | 0.575 | 0.016 | 0.512 |
|  | PLS | 0.563 | 0.499 | 0.205 | 0.793 | -0.003 | 0.494 |
|  | RF | 0.607 | 0.506 | 0.048 | 0.964 | 0.030 | 0.516 |
|  | rpart | 0.604 | 0.499 | 0.024 | 0.974 | -0.005 | 0.961 |
|  | SVM | 0.610 | 0.500 | 1.000 | 0.000 | 0.000 | 0.493 |
|  | XGB | 0.598 | 0.508 | 0.104 | 0.913 | 0.028 | 0.508 |
| MACCS | GLM | 0.828 | 0.810 | 0.731 | 0.889 | 0.634 | 0.888 |
|  | KNN | 0.846 | 0.823 | 0.718 | 0.929 | 0.674 | 0.945 |
|  | MLP | 0.837 | 0.824 | 0.763 | 0.884 | 0.655 | 0.901 |
|  | PLS | 0.818 | 0.795 | 0.686 | 0.903 | 0.613 | 0.883 |
|  | RF | 0.876 | 0.863 | 0.809 | 0.918 | 0.736 | 0.928 |
|  | rpart | 0.785 | 0.749 | 0.582 | 0.915 | 0.541 | 0.902 |
|  | SVM | 0.867 | 0.855 | 0.801 | 0.910 | 0.719 | 0.921 |
|  | XGB | 0.863 | 0.852 | 0.801 | 0.903 | 0.710 | 0.922 |
| Pubchem | GLM | 0.852 | 0.834 | 0.755 | 0.913 | 0.685 | 0.916 |
|  | KNN | 0.829 | 0.806 | 0.702 | 0.910 | 0.635 | 0.947 |
|  | MLP | 0.849 | 0.842 | 0.814 | 0.871 | 0.683 | 0.915 |
|  | PLS | 0.841 | 0.822 | 0.734 | 0.910 | 0.662 | 0.902 |
|  | RF | 0.873 | 0.863 | 0.814 | 0.912 | 0.732 | 0.930 |
|  | rpart | 0.815 | 0.788 | 0.665 | 0.912 | 0.606 | 0.932 |
|  | SVM | 0.865 | 0.857 | 0.819 | 0.895 | 0.716 | 0.920 |
|  | XGB | 0.858 | 0.846 | 0.790 | 0.901 | 0.699 | 0.920 |
| RDK5 | GLM | 0.559 | 0.513 | 0.303 | 0.723 | 0.028 | 0.536 |
|  | KNN | 0.560 | 0.522 | 0.346 | 0.697 | 0.045 | 0.619 |
|  | MLP | 0.559 | 0.538 | 0.444 | 0.633 | 0.077 | 0.580 |
|  | PLS | 0.573 | 0.511 | 0.229 | 0.793 | 0.025 | 0.512 |
|  | RF | 0.588 | 0.533 | 0.282 | 0.784 | 0.075 | 0.583 |
|  | rpart | 0.607 | 0.498 | 0.003 | 0.993 | -0.028 | 0.976 |
|  | SVM | 0.606 | 0.498 | 0.005 | 0.990 | -0.026 | 0.545 |
|  | XGB | 0.550 | 0.489 | 0.213 | 0.765 | -0.026 | 0.511 |

**Table S5** Independent test results of 72 base-classifiers developed using eight different ML algorithms and nine molecular descriptors for ERβ.

| **Descriptor** | **Method** | **ACC** | **BACC** | **Sn** | **Sp** | **MCC** | **AUC** |
| --- | --- | --- | --- | --- | --- | --- | --- |
| AP2D | GLM | 0.819 | 0.789 | 0.653 | 0.926 | 0.616 | 0.857 |
|  | KNN | 0.790 | 0.773 | 0.695 | 0.851 | 0.554 | 0.933 |
|  | MLP | 0.798 | 0.782 | 0.705 | 0.858 | 0.572 | 0.854 |
|  | PLS | 0.823 | 0.804 | 0.716 | 0.892 | 0.623 | 0.880 |
|  | RF | 0.807 | 0.775 | 0.632 | 0.919 | 0.588 | 0.869 |
|  | rpart | 0.774 | 0.731 | 0.537 | 0.926 | 0.518 | 0.927 |
|  | SVM | 0.774 | 0.752 | 0.653 | 0.851 | 0.517 | 0.821 |
|  | XGB | 0.802 | 0.779 | 0.674 | 0.885 | 0.578 | 0.855 |
| AP2DC | GLM | 0.807 | 0.773 | 0.621 | 0.926 | 0.589 | 0.857 |
|  | KNN | 0.774 | 0.741 | 0.589 | 0.892 | 0.514 | 0.925 |
|  | MLP | 0.844 | 0.819 | 0.705 | 0.932 | 0.669 | 0.905 |
|  | PLS | 0.819 | 0.799 | 0.705 | 0.892 | 0.614 | 0.899 |
|  | RF | 0.864 | 0.845 | 0.758 | 0.932 | 0.712 | 0.914 |
|  | rpart | 0.774 | 0.733 | 0.547 | 0.919 | 0.517 | 0.895 |
|  | SVM | 0.798 | 0.780 | 0.695 | 0.865 | 0.571 | 0.862 |
|  | XGB | 0.864 | 0.847 | 0.768 | 0.926 | 0.712 | 0.898 |
| FP4 | GLM | 0.798 | 0.768 | 0.632 | 0.905 | 0.569 | 0.848 |
|  | KNN | 0.815 | 0.790 | 0.674 | 0.905 | 0.605 | 0.924 |
|  | MLP | 0.827 | 0.807 | 0.716 | 0.899 | 0.632 | 0.844 |
|  | PLS | 0.856 | 0.837 | 0.747 | 0.926 | 0.695 | 0.898 |
|  | RF | 0.844 | 0.819 | 0.705 | 0.932 | 0.669 | 0.891 |
|  | rpart | 0.798 | 0.763 | 0.600 | 0.926 | 0.571 | 0.927 |
|  | SVM | 0.840 | 0.825 | 0.758 | 0.892 | 0.660 | 0.866 |
|  | XGB | 0.802 | 0.778 | 0.663 | 0.892 | 0.578 | 0.828 |
| FP4C | GLM | 0.827 | 0.798 | 0.663 | 0.932 | 0.634 | 0.876 |
|  | KNN | 0.831 | 0.812 | 0.726 | 0.899 | 0.641 | 0.967 |
|  | MLP | 0.827 | 0.815 | 0.758 | 0.872 | 0.635 | 0.872 |
|  | PLS | 0.840 | 0.814 | 0.695 | 0.932 | 0.660 | 0.917 |
|  | RF | 0.860 | 0.840 | 0.747 | 0.932 | 0.704 | 0.925 |
|  | rpart | 0.782 | 0.738 | 0.537 | 0.939 | 0.539 | 0.870 |
|  | SVM | 0.848 | 0.830 | 0.747 | 0.912 | 0.677 | 0.902 |
|  | XGB | 0.819 | 0.795 | 0.684 | 0.905 | 0.614 | 0.892 |
| KR | GLM | 0.543 | 0.495 | 0.274 | 0.716 | -0.011 | 0.501 |
|  | KNN | 0.572 | 0.524 | 0.305 | 0.743 | 0.053 | 0.594 |
|  | MLP | 0.477 | 0.445 | 0.295 | 0.595 | -0.112 | 0.423 |
|  | PLS | 0.564 | 0.504 | 0.232 | 0.777 | 0.010 | 0.538 |
|  | RF | 0.609 | 0.504 | 0.021 | 0.986 | 0.029 | 0.525 |
|  | rpart | 0.609 | 0.500 | 1.000 | 0.000 | 0.000 | 1.000 |
|  | SVM | 0.609 | 0.500 | 1.000 | 0.000 | 0.000 | 0.468 |
|  | XGB | 0.601 | 0.501 | 0.042 | 0.959 | 0.004 | 0.518 |
| KRC | GLM | 0.543 | 0.472 | 0.147 | 0.797 | -0.070 | 0.496 |
|  | KNN | 0.556 | 0.516 | 0.337 | 0.696 | 0.034 | 0.619 |
|  | MLP | 0.527 | 0.506 | 0.411 | 0.601 | 0.012 | 0.512 |
|  | PLS | 0.597 | 0.526 | 0.200 | 0.851 | 0.067 | 0.535 |
|  | RF | 0.605 | 0.510 | 0.074 | 0.946 | 0.040 | 0.507 |
|  | rpart | 0.609 | 0.500 | 1.000 | 0.000 | 0.000 | 1.000 |
|  | SVM | 0.609 | 0.500 | 1.000 | 0.000 | 0.000 | 0.469 |
|  | XGB | 0.572 | 0.479 | 0.053 | 0.905 | -0.076 | 0.486 |
| MACCS | GLM | 0.802 | 0.781 | 0.684 | 0.878 | 0.579 | 0.844 |
|  | KNN | 0.786 | 0.756 | 0.621 | 0.892 | 0.542 | 0.933 |
|  | MLP | 0.831 | 0.809 | 0.705 | 0.912 | 0.641 | 0.855 |
|  | PLS | 0.840 | 0.817 | 0.716 | 0.919 | 0.659 | 0.918 |
|  | RF | 0.856 | 0.835 | 0.737 | 0.932 | 0.695 | 0.914 |
|  | rpart | 0.798 | 0.759 | 0.579 | 0.939 | 0.574 | 0.926 |
|  | SVM | 0.831 | 0.807 | 0.695 | 0.919 | 0.641 | 0.881 |
|  | XGB | 0.819 | 0.800 | 0.716 | 0.885 | 0.615 | 0.895 |
| Pubchem | GLM | 0.840 | 0.812 | 0.684 | 0.939 | 0.661 | 0.888 |
|  | KNN | 0.811 | 0.775 | 0.611 | 0.939 | 0.600 | 0.932 |
|  | MLP | 0.831 | 0.811 | 0.716 | 0.905 | 0.641 | 0.889 |
|  | PLS | 0.889 | 0.871 | 0.789 | 0.953 | 0.766 | 0.937 |
|  | RF | 0.835 | 0.810 | 0.695 | 0.926 | 0.651 | 0.915 |
|  | rpart | 0.802 | 0.774 | 0.642 | 0.905 | 0.578 | 0.920 |
|  | SVM | 0.844 | 0.826 | 0.747 | 0.905 | 0.668 | 0.881 |
|  | XGB | 0.840 | 0.817 | 0.716 | 0.919 | 0.659 | 0.852 |
| RDK5 | GLM | 0.531 | 0.474 | 0.211 | 0.736 | -0.060 | 0.482 |
|  | KNN | 0.543 | 0.516 | 0.389 | 0.642 | 0.032 | 0.584 |
|  | MLP | 0.449 | 0.442 | 0.411 | 0.473 | -0.114 | 0.466 |
|  | PLS | 0.543 | 0.491 | 0.253 | 0.730 | -0.020 | 0.478 |
|  | RF | 0.494 | 0.453 | 0.263 | 0.642 | -0.099 | 0.453 |
|  | rpart | 0.609 | 0.500 | 1.000 | 0.000 | 0.000 | 1.000 |
|  | SVM | 0.609 | 0.500 | 1.000 | 0.000 | 0.000 | 0.487 |
|  | XGB | 0.527 | 0.463 | 0.168 | 0.757 | -0.089 | 0.466 |
